# Process evaluation of a randomised controlled trial intervention designed to improve rehabilitation services for Aboriginal Australians after brain injury: the Healing Right Way Trial

**DOI:** 10.1186/s12913-024-11390-5

**Published:** 2024-08-20

**Authors:** Judith M. Katzenellenbogen, Jane White, Melanie Robinson, Sandra C. Thompson, Amy Epstein, Mandy Stanley, Jane Klobas, Emma Haynes, Elizabeth A. Armstrong, Juli Coffin, Rachel Skoss

**Affiliations:** 1 Cardiovascular Epidemiology Research Centre, School of Population and Global Health, The University of Western Australia, Stirling Highway, Nedlands, Perth, WA 6009 Australia; 2 School of Medical and Health Science, Edith Cowan University, Perth, Australia; 3 Department of Health of Western Australia, Child and Adolescent Health Service, Perth, Australia; 4 Murdoch University, Perth, Australia; 5 Western Australian Centre for Rural Health, The University of Western Australia, Geraldton, Australia; 6 University Centre for Rural Health South West, Edith Cowan University, Bunbury, Australia; 7 Institute for Health Research, University of Notre Dame Australia, Perth, Australia; 8 Telethon Kids Institute, Perth, WA Australia

**Keywords:** Process evaluation, Indigenous health, Acquired brain injury, Cultural security, Rehabilitation, Australia

## Abstract

**Background:**

Healing Right Way (HRW) aimed to improve health outcomes for Aboriginal Australians with stroke or traumatic brain injury by facilitating system-level access to culturally secure rehabilitation services. Using a stepped-wedge randomised controlled trial (RCT) design (ACTRN12618000139279, 30/01/2018), a two-pronged intervention was introduced in four rural and four urban hospitals, comprising 1.Cultural security training (CST) for staff and 2.Training/employment of Aboriginal Brain Injury Coordinators (ABIC) to support Aboriginal patients for 6-months post-injury. Three-quarters of recruited patients lived rurally. The main outcome measure was quality-of-life, with secondary outcomes including functional measures, minimum processes of care (MPC); number rehabilitation occasions of service received, and improved hospital experience. Assessments were undertaken at baseline, 12- and 26-weeks post-injury. Only MPCs and hospital experience were found to improve among intervention patients.

We report on the process evaluation aiming to support interpretation and translation of results.

**Methods:**

Using mixed methods, the evaluation design was informed by the Consolidated Framework for Implementation Research. Data sources included minutes, project logs, surveys, semi-structured interviews, and observations.

Four evaluation questions provided a basis for systematic determination of the quality of the trial. Findings from separate sources were combined to synthesise the emerging themes that addressed the evaluation questions. Three components were considered separately: the trial process, CST and ABIC.

**Results:**

The complex HRW trial was implemented to a satisfactory level despite challenging setting factors, particularly rural–urban system dynamics. Patient recruitment constraints could not be overcome. The vulnerability of stepped-wedge designs to time effects influenced recruitment and trial results, due to COVID. Despite relatively high follow-up, including to rural/remote areas, data points were reduced. The lack of culturally appropriate assessment tools influenced the quality/completeness of assessment data. The ABIC role was deemed feasible and well-received. The CST involved complex logistics, but rated highly although online components were often incomplete. Project management was responsive to staff, patients and setting factors.

**Conclusions:**

Despite mostly equivocal results, the ABIC role was feasible within mainstream hospitals and the CST was highly valued. Learnings will help build robust state-wide models of culturally secure rehabilitation for Aboriginal people after brain injury, including MPC, workforce, training and follow-up.

**Supplementary Information:**

The online version contains supplementary material available at 10.1186/s12913-024-11390-5.

## Background

Aboriginal and Torres Strait Islander (hereafter respectfully ‘Aboriginal’) peoples, Australia’s First Nations peoples, comprise 3.3% of the population of Western Australian (WA), and are culturally, linguistically, and socio-economically diverse. In 2016, 62% of Aboriginal people in WA lived outside of the Perth metropolitan region. Aboriginal culture remains a source of strength, despite ongoing effects of colonisation, including disparities in health [1].

Access to rehabilitation services following stroke and traumatic brain injury (TBI) is poorer for Aboriginal people [2–5], despite high incidence [6–8]. Geographical, logistical, and cultural barriers often impact recovery and functional outcomes, with challenges exacerbated for people living rurally. Previous studies [7, 8] have documented over 80% of Aboriginal people living with stroke or TBI lived rurally at the time of their injury, with over 50% being from remote or very remote areas. Given that Aboriginal peoples’ interaction with health services is frequently marred by systemic barriers, the imperative for health services that address Aboriginal peoples’ needs and respond to Aboriginal calls for change is reflected in Australian government policy [9].

The Healing Right Way (HRW) randomised controlled trial (RCT) [10, 11] (ACTRN12618000139279, registered 30/01/2018) was developed in response to low representation of Aboriginal people in brain injury rehabilitation services and recommendations from Aboriginal consumers and their families to improve the cultural security of services [2]. Cultural security aims to provide cultural safety for patients; it is attained when institutions, services and their staff have awareness of Aboriginal cultural values, practices and world views and implement this knowledge into policy and practice to address patient needs [12]. Undertaken between 2017 and 2022, HRW focussed on enhancing rehabilitation services and quality of life for Aboriginal Australians in WA following stroke and TBI, based within an Aboriginal Research framework [13] and incorporating principles from Indigenous Standpoint Theory [14]. Recognising the need for a decolonising perspective, Aboriginal peoples’ experiences, recommendation and leadership in the research process are central to this approach, and has been applied previously to disability in an Aboriginal context [15, 16]. Using a stepped-wedge design, a two-pronged intervention was introduced sequentially in four rural and four metropolitan hospitals, aiming to improve health outcomes by facilitating access to interdisciplinary and culturally secure rehabilitation services for Aboriginal people with stroke/TBI, thereby providing a robust best practice model [10, 11] (Table 1).

**Table 1:** Crucial overview of HRW trial design, as implemented, and conclusions of statistical results

**Study design**
Healing Right Way was a stepped-wedge cluster randomised controlled trial (RCT) with four steps. The intervention was rolled out to one metropolitan and one regional site per step. Control (non-intervention) data were collected from patients recruited at each hospital site for a minimum of 12 months prior to roll out. All sites received the intervention for a minimum of 12 months
**Patient Recruitment**
Aboriginal adults, admitted to hospital for acquired brain injury (ABI) resulting from stroke or traumatic brain injury (TBI), were identified by Research Site Contacts (RSC) and recruited Eligibility criteria included: • Identification as Aboriginal • ≥ Age 18 years • Acute ischaemic or haemorrhagic stroke or acute TBI • Neurological deficit present as reflected in NIHSS > 0 • Able to benefit from rehabilitation as determined by the medical and allied health team Between 2018 and 2021, 108 patients (75% rural/remote residents) were recruited to the study (82% stroke, 18% TBI), 47 in the control and 61 intervention groups respectively
**Intervention**
The two-pronged intervention comprised of: 1. Training and employment of region-based Aboriginal Brain Injury Coordinators (ABIC) to support Aboriginal people with ABI and their families for 6-months post injury 2. Cultural security training (face-to-face and online formats) targeting hospital staff and encompassing aspects of care specific to Aboriginal people with brain injury
**Data collection**
Multiple assessment tools and questionnaires standardised to the general population were used by assessors with nursing and allied health backgrounds to collect data relevant to participant and service outcomes, and to the economic impact of injury and costing of the intervention (see outcomes below) Baseline data were collected by baseline assessors within 6 weeks of injury. Blinded assessors collected follow-up data within 12 weeks and 26 weeks post injury
**Outcome measures and results of RCT**
The primary outcome measure related to quality of life as measured on the EuroQOL-5D-3L VAS [17] at 6-months post injury – **Result: no significant difference between groups** Secondary outcome measures related to: • Severity of disability (Modified Rankin Scale [18]) – **no significant difference,** • Functional independence (Functional Independence Measure—FIM [19]) – **no significant difference**, • Anxiety and depression (Hospital Anxiety and Depression Scale—HADS) – **no significant difference**, • Caregiver strain (Modified Carer Strain Index [20]) – **no significant difference,** • Clinical service provision (compliance with Minimum Process of Care indicators—MPC) – **significantly higher in the intervention group**, • Service utilisation (Occasions of Service—OoS) – **no** **significant difference**, and • Patient experiences (Participant Survey) – **greater satisfaction with hospital services in the intervention group**

The two components of the intervention were *training and employment of region-based Aboriginal Brain Injury Coordinators (ABIC)* (See Table 2) and *cultural security training (CST) for hospital staff* (See Table 3)*.* Identified by hospital-employed Research Site Contacts (RSCs), in-patients were recruited in participating hospitals, with Baseline Assessors obtaining consent and collecting baseline data. Blinded Assessors followed up participating patients and collected outcome data at 12- and 26-weeks post-brain injury [10], with data captured in a REDCap database. A university-based HRW management team, including the chief investigator, project co-ordinator, data manager and ABIC trainer/supervisor oversaw the logistics of the trial with input where needed from Aboriginal investigators and partners.

**Table 2:** Description of Aboriginal Brain Injury Coordinator intervention

The ABIC role employed an Aboriginal person for one day per week at each project site once they entered the intervention phase of the study. The ABIC role centred on supporting participants and their family/carers for six months after the brain injury. The aim was to have a positive impact on participants’ overall health and wellbeing, focusing on education, support, liaison, and advocacy. ABICs were based with different employers (hospital, local community controlled Aboriginal Medical Service (AMS), or offices of the Neurological Council of Western Australia (a community neurological nursing service)), depending on site preference. A minimum of six ABIC contacts with the participant was planned up to 26 weeks post-injury.

**Table 3:** Description of the CST intervention

The CST targeted multiple disciplines and seniority levels in the hospital system and involved a three-hour face-to-face workshop (at some sites three one-hour sessions were conducted), and three online modules to be completed within four weeks of the workshop. The training was clinically focused and centred on definitions of cultural security, a holistic Aboriginal model of health, and communication through clinical yarning. It included case studies of Aboriginal people with ABI supplemented with videos of people discussing their experiences since their event, as well as discussion of local contexts and cultural security in their workplaces. The sessions were co-facilitated by a HRW clinician and an Aboriginal person from or with knowledge of the local Aboriginal context. HRW aimed to train at least 20 people at each site with training to be offered every six months to accommodate staff turnover.	

HRW was a complex intervention trial [10]. The interventions involved different types of health service providers, including government and non-government, in-patient and community-based; eight hospital sites (four metropolitan and four regional); two different brain injury patient groups (stroke/TBI); and two distinct interventions which commenced simultaneously at each hospital site but were rolled out to different hospitals in different trial steps. Multiple formal partners, including governmental, non-governmental and Aboriginal community-controlled health services, were engaged in the establishment and maintenance of the trial administrative processes as well as the interventions.

Given the complexity of the intervention, a process evaluation was a critical component [21, 22]. The value of process evaluations lies in their ability to assess implementation, clarify underlying mechanisms, and identify contextual factors associated with variation in study outcomes [23]. Understanding the challenges and opportunities in implementing programmes has the potential to substantially improve the success of health interventions. As outlined in our protocol paper [24], a mixed methods process evaluation with both prospective and retrospective data collection was undertaken, nested within the parent HRW trial.

Armstrong et al. [11] reported on the results of the trial which were equivocal on the primary outcome (increased quality-of-life) and most secondary outcomes, although showed improvements in achievement of minimum processes of care (MPC) for the intervention group of patients. Surveys with patients at 26-weeks post-injury found that those in the intervention group were more satisfied with hospital services than those in the control group.

In this paper, we report on the process evaluation of HRW which aimed to support interpretation of the outcomes and explanation of the study results. The focus on rural issues raised in the study constitutes essential service information for this population who are predominantly rurally based.

## Methods

### Evaluation design and framework

The overall design of the process evaluation was informed by the original Consolidated Framework for Implementation Research (CFIR) [25], which guided data collection, analysis, and reporting of findings.

The CFIR framework was selected because of its ability to accommodate multiple interventions, and to provide continuity between the research and its translation/implementation goals. The CFIR recognition of contextual factors allowed a pragmatic approach to evaluation in a complex and often disorganised real-world setting. This framework has been used in other health-related [26] and Aboriginal contexts [27] and is useful to guide rapid cycle evaluation to systematically identify where adjustments and refinements can be made during implementation, meeting our objective to inform/refine the HRW intervention.

The HRW process evaluation considered all five major domains of CFIR, namely *Outer setting* (broader setting/context); *Inner setting* (in which the intervention is being implemented); *Intervention characteristics* (features of the intervention); *Individual*s (roles and characteristics of individuals involved); and *Implementation/process* (activities/strategies used to implement the intervention). Although referred to, the more detailed ‘constructs’ for each domain were not specifically drawn on.

Four key evaluation questions were developed to provide a basis for systematic determination of the quality of the HRW trial [28]. Each question was mapped to one or more of the CFIR domains and the questions were refined as the trial progressed to best capture the most relevant aspects of the trial. Three components of HRW were considered separately: the trial process (including research design and project management) and each of the two interventions (CST and ABIC) (Table 4).

**Table 4:** Mapping of evaluation questions and relevant data sources to CFIR domains

			**CFIR domains**				
**HRW component**	**Evaluation questions**	**Data sources**	**Outer**	**Inner**	**Intervention**	**Individuals**	**Implementation**
Trial Processes	1. To what extent were the trial processes (research approaches and project management) implemented as planned?	Project Log, Key project staff interviews, Project and partner meeting minutes, Observations	x	x	x	x	x
Intervention: ABIC	1. How did contextual complexity affect the implementation of the ABIC role and how was this managed?	Key HRW project staff interviews, Project log, Project and partner meeting minutes, ABIC training log, ABIC interviews	x	x		x	x
	2. What were the factors influencing the implementation of the ABIC role and how were these managed?	Key project staff interviews, Project log, Project and partner meeting minutes, ABIC interviews	x	x	x	x	x
	3. To what extent was the ABIC service delivered as planned?	ABIC interviews, Key project staff interviews, Project log	x	x		x	x
	4. How effective was the ABIC role?	ABIC interviews, Key project staff interviews, Project Log	x	x	x	x	x
Intervention: Culturally Secure Training	1. How did contextual complexity affect the CST implementation and how was this managed?	Project Log, Key project staff interviews, Cultural security training surveys	x	x			x
	2. What were the factors influencing the implementation of the CST and how were these managed?	Project log, Key project staff interviews, Project and partner meeting minutes	x	x	x	x	x
	3. To what extent was the training delivered as planned?	Attendance Log, Cultural security training surveys, Key project staff interviews, Observations	x	x		x	x
	4. How effective was the training?	Cultural security training surveys, Key project staff interviews, Participant/patient experience surveys, Observations	x	x	x	x	x

### Data collection

The data sources included meeting minutes, a detailed project log, surveys, semi-structured interviews, and CST observations, with interview questions, questionnaires and data gathering structured to ensure CFIR domains were addressed (see interview guides and questionnaires in supplement). Both quantitative and qualitative data were predominantly collected prospectively from university-based research staff (*n* = 4 staff, multiple times), CST attendees (201 of 250 participants), trial participants (*n* = 108 patients) and administrative records during HRW. Assessors (*n* = 8), RSCs (*n* = 7) and ABICs (*n* = 6 of 9) were interviewed at the end of the trial. Some sources were relevant to all three evaluation components, and some only to specific aspects. Timing of data collection and detailed methods for each data source are outlined in the protocol [24] (also see Supplementary Fig. 1). Although we recognise that consumers of rehabilitation services are not always considered ‘patients’, for the purpose of this evaluation paper, we mostly refer to them as patient participants (or patients) to differentiate them from the other types of participants within the evaluation [29].

### Data analysis

**Fig. 1:**
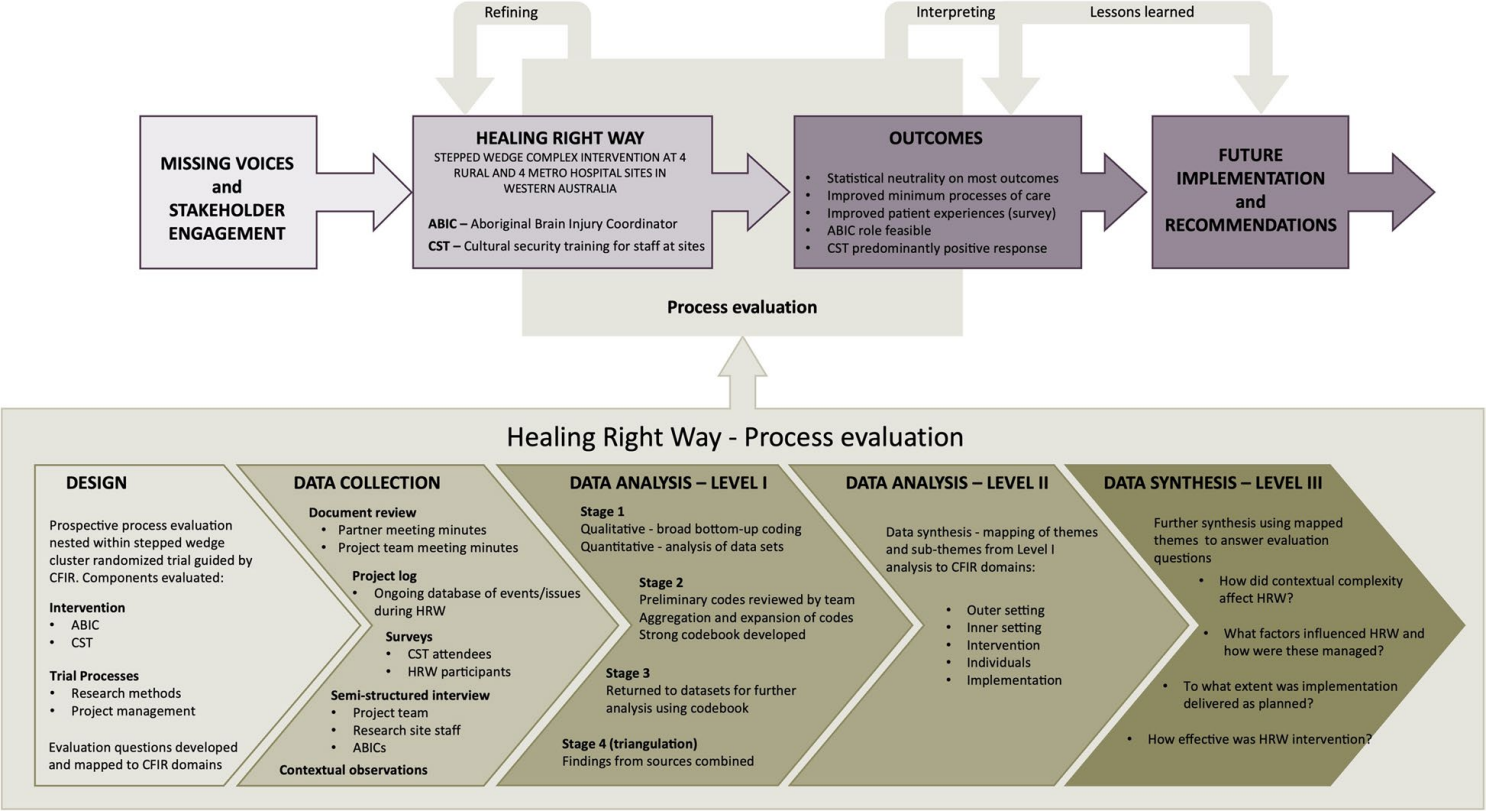
Schematic overview of the evolution of Healing Right Way, the role of the process evaluation and its methods

Interim analyses of prospectively collected data from the project log, management team interviews and meeting minutes were summarised in quarterly reports during the conduct of HRW and fed back to the management and investigator team of HRW to inform trial implementation. The analysis synthesising all data sources from all time periods and reported in this paper was carried out at three levels at the end of the trial (see Fig. 1): I – initial analysis; II – mapping to CFIR domains; III – further synthesis to answer the evaluation questions and enhance understanding/interpretation of the key results from the statistical analysis of the HRW trial outcomes.

Level I analysis was done in four stages. In Stage 1, different members of the evaluation team examined the separate data sets (e.g. CST surveys, project log, research staff interviews, ABIC interviews). For qualitative data from all sources other than patient surveys, we took a deductive approach to coding of themes [30], guided by the CFIR. In Stage 2, preliminary codes were reviewed by the broad team, with aggregation and expansion of codes to yield a comprehensive codebook. Stage 3 involved individual members of the team consulting this refined codebook in more detailed analysis of the separate datasets. Further expansion/refinement of codes occurred through discussion within the team, as necessary. An external researcher, blinded to patient group separately analysed patient surveys. In Stage 4, findings from separate sources were combined to assess the extent to which they supported or contradicted each other (triangulation) and to develop an overall understanding of the emerging themes and sub-themes.

During Level II analysis, Level I themes and sub-themes for each of the three components (Trial Process, ABIC, and CST) were mapped, tabulated and synthesised according to the CFIR domains, guided by the 2009 and 2022 domain definitions [25, 31]. After piloting our analytic approach, analysis at the detailed construct level was not undertaken specifically but guided our mapping of themes to domains. This was done to reduce complexity and rigidity, and add value to ongoing implementation of the RCT. All data could be mapped to existing domains. Themes relevant to more than one CFIR domain were mapped accordingly. The Trial Processes were further evaluated according to different methodological aspects of the research design (e.g. study design, participant recruitment) and components of project management (e.g. staff appointments, training/support). Constituting Level III data synthesis, the evaluation questions (refined from original questions) for the different components of the study were answered using the existing mapped themes.

### Evaluation team

The evaluation team was co-led by an external evaluation expert (RS) and one of the chief investigators of the HRW trial (JMK). An external Aboriginal policy/researcher (MR), as well as three other HRW investigators (SCT, BA including one Aboriginal, JC) contributed to the core evaluation team. One researcher (JW) was embedded in the HRW management team for 1–2 days week, with two other external researchers (AE, JK) conducting specific sub-studies. Aboriginal members of the process evaluation team guided the cultural lens of the evaluation including design of research questions, data collection methods, data analysis and synthesis.

### Data availability

Neither of the datasets from the current study or the parent study (HRW) are available as per Indigenous data sovereignty requirements.

## Results

**Fig. 2:**
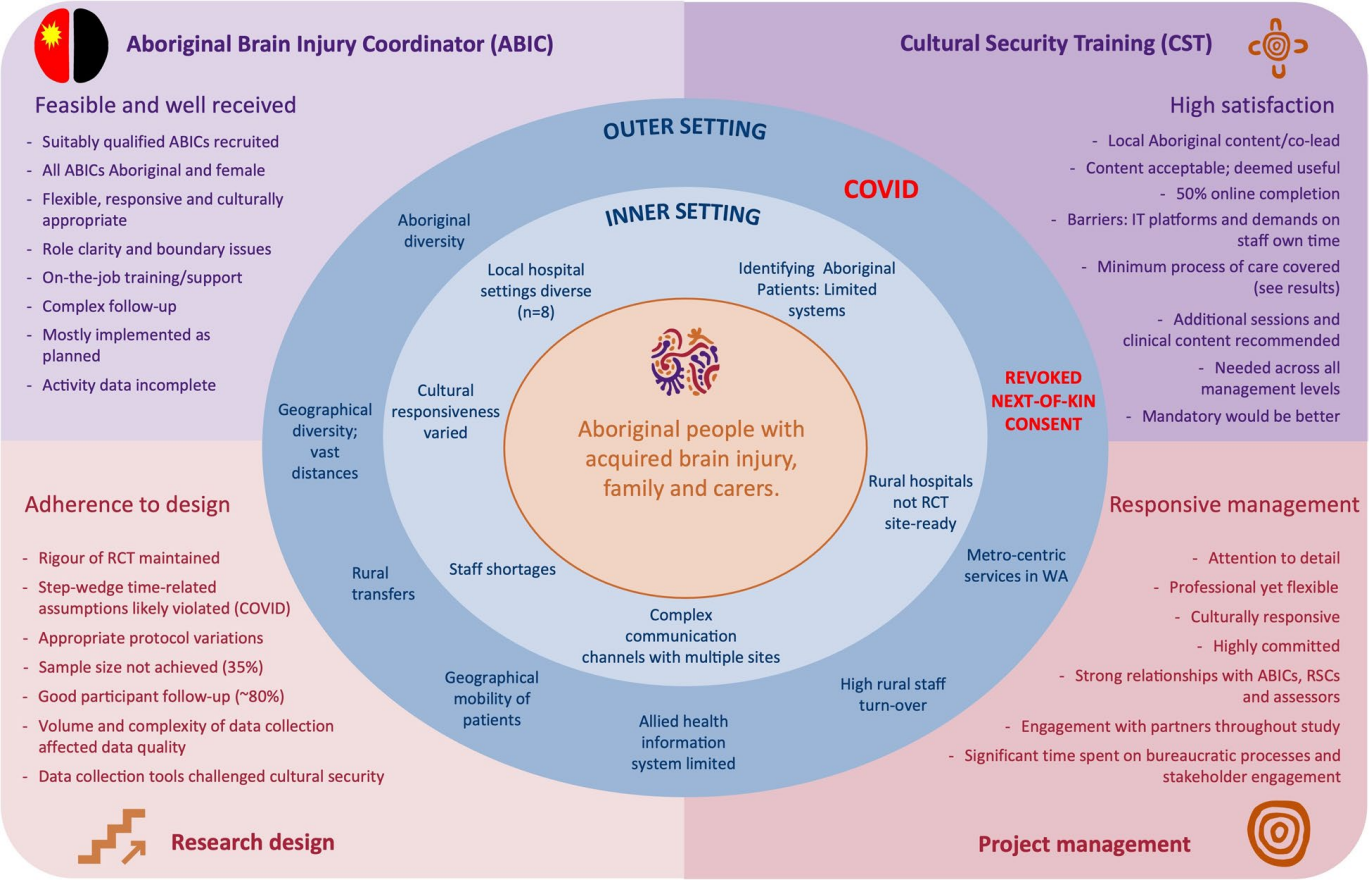
Summary themes emerging from process evaluation of Healing Right Way, by trial component

Figure 2 summarises the findings, with trial process evaluation results represented in the lower half of the diagram (bottom left hand side (LHS): research design; bottom right hand side (RHS): project management), and ABIC and CST intervention and implementation process results in the top left and top right quadrants, respectively. The concentric circles in the centre of the diagram represent the CFIR contextual domains which cut across all processes: the outer setting, inner setting and individuals (the diagram emphasises the patients and their carers; in the results, we consider all individuals involved in HRW).

This section begins with an introduction to factors in the three contextual domains which cut across all components of HRW. Separate review of each trial component follows, addressing the evaluation questions outlined in Table 4. The results represent the synthesis of findings across all data sources and direct quotes from the data are provided to support the narrative. The third evaluation question (extent to which each component was successfully implemented) is summarised in Table 6 (trial design), 7 (ABIC intervention), and 8 (CST intervention). Detailed summaries of the separate sub-studies will be published as separate papers. Supplementary Tables 2 and 3 provide a tabulated summary of findings by CFIR domain.

### Overall contextual complexities affecting HRW

#### Outer setting

Outer setting factors, including WA’s geographical vastness and sparse population outside metropolitan areas greatly influenced HRW. Important factors were access of rural patients to the specialist acute hospitals almost exclusively based in metropolitan areas, requiring emergency transfer of patients from regional areas, sparse availability of rehabilitation services in rural areas, acute workforce shortages in regional and remote areas, too few allied health staff to service large geographical regions, high health service staff turnover, and relatively few Aboriginal health professionals. In addition, two major unforeseen complexities in the outer setting reverberated through the study. Policy change in 2018 resulted in health ethics/governance committees revoking permission for research studies to use next-of-kin consent for recruitment of those unable to consent [32, 33]. The COVID-19 pandemic was declared mid-way through HRW, and multiple adverse health, social and economic impacts were experienced Australia-wide. For many Aboriginal Australians, this meant exacerbation of poor socio-economic and environmental conditions, limited community/family contact due to requirements to isolate, and disrupted services and food supply chains. Importantly, crowded housing conditions were magnified when many people returned to non-metropolitan regions during lockdowns [34]. Stroke presentations and imaging [35] reduced, reflecting wider avoidance of hospitals [36]. Diversion of health resources to manage the pandemic had a negative impact on stroke care [37, 38]. While showing tremendous leadership in tackling COVID, Aboriginal Medical Services were put under extreme strain [39–41]. These major disruptions to services required logistical adaptations in HRW implementation while staying true to the trial design.

#### Inner setting

At the inner setting level, trial logistics were further challenged by greater diversity than expected in systems and processes across hospitals with respect to leadership, data access, staff recruitment, models of care, employment conditions, training, research readiness, and requirements for project staff working. Communication channels within sites were often challenging, exacerbated by senior staff turnover. The different settings across the eight sites required tailoring of some trial processes to specific site needs, including processes to identify Aboriginal patients and shaping cultural training for the local Aboriginal context. Places of employment for ABICs and, thus, access to patients varied by region. Ensuring the cultural security of the project for participants and staff was challenged by systemic barriers and differences in cultural responsiveness across sites.

#### Individuals

Individual domain challenges included recruitment of appropriately skilled staff to participate in HRW interventions and research processes, particularly in rural areas. Cultural factors affected patient recruitment and follow-up with some potential participants feeling distress and unfamiliarity with being away from Country, while also experiencing brain injury effects. Over 70% of HRW patient participants were rural residents recruited in metropolitan hospitals, complicating follow-up.

### Trial processes (Fig. 2, lower LHS)

Two components of the trial processes were analysed separately: implementation of the research design (including processes) and project management.

#### Research design and processes

Despite outer and inner setting challenges to implementation of the research design, evaluation of the trial processes shows that the rigour of the RCT was maintained, with elements implemented as planned or appropriately adapted while also remaining within the RCT requirements and protocol. In this section, we outline contextual challenges to trial design, sample, and data collection along with variations in processes made to maintain the rigour of the RCT.

### Trial design

Sequential implementation of the interventions across the eight hospital sites according to the stepped-wedge design was achieved, although the first (control) step was extended by 6 months (Table 5). However, a major assumption of stepped wedge designs–that no factors in calendar time affect the outcome–was violated with the onset of COVID-19. While most control patient participants (94%) were recruited pre-pandemic, almost all intervention participants (84%) were recruited after restrictions had been introduced. The different conditions pre- and post-COVID-19 restrictions likely confounded results, including the primary outcome, quality of life.

**Table 5:**
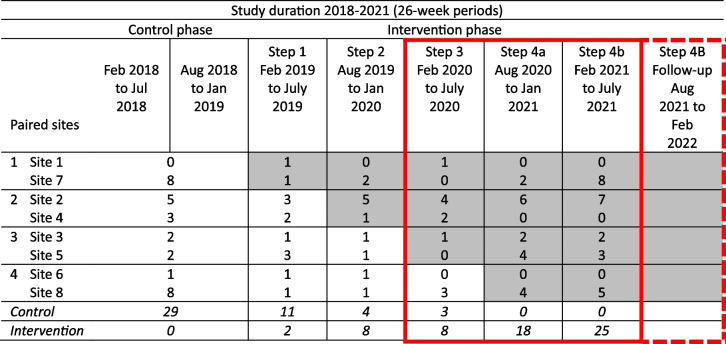
HRW stepped-wedge design for patient recruitment, as implemented

*Source*: Adapted from Armstrong B, et al. Healing Right Way: Study protocol for a randomised control trial to enhance rehabilitation services and improve quality of life in Aboriginal Australians after brain injury ^10^

### Recruitment

**Fig. 3:**
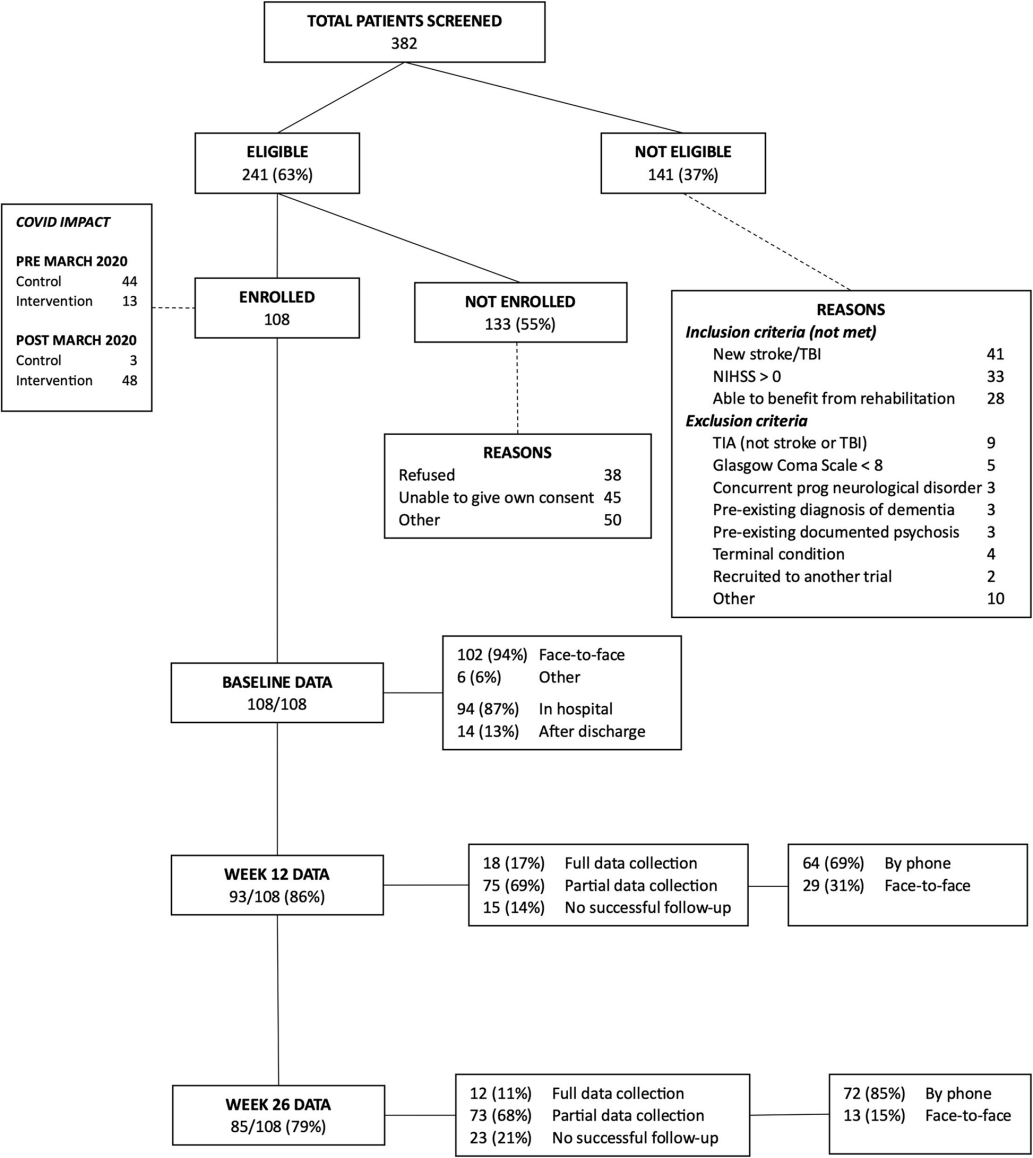
Flow chart of patients recruited to Healing Right Way and Assessment data collected

Patient recruitment into the trial (Fig. 3) followed the trial protocol. However, recruitment and follow-up were negatively affected by several of the contextual factors described earlier. Timing of recruitment had to consider patients’ circumstances, with many rural patients off-Country in unfamiliar hospital environments and away from family.

COVID-19 likely impacted patient recruitment through a reduced pool of eligible patients and reduced Assessor ability to enter hospitals and make face-to-face contact.

We noticed just a general decrease in stroke and head injury referrals coming in... And I think that was because people weren't presenting to hospital because they were fearful. (Regional RSC)

The patient pool was further limited by the requirement for wet-ink patient consent of family or rapidly discharged patients. Finances, commitments, distance, and COVID-19 curtailed family travel, limiting family participation in recruitment.

Identification of eligible patients was often difficult. The capacity of non-Aboriginal RSCs to identify potential participants for recruitment varied, with work pressures exacerbated by COVID-19:

patients could be more guarded with me coming and asking about joining our study… I felt like that was better if I had the Aboriginal Liaison Officer involved. (Metro RSC)

Difficulties applying eligibility criteria arose when multiple comorbidities complicated diagnosis. TBI recruitment was underachieved, reflecting the distressing circumstances around injury, rapid discharge of potentially eligible patients with milder injuries, and challenges in interpretation of TBI diagnostic terminology.

Inevitably, some potential participants (*n* = 38) declined to participate. Slow patient recruitment delayed the start of the trial by six months. Ethics amendments permitted changes to recruitment processes, including extension of the first control step, ability to enrol via verbal consent, expansion of the recruitment window from four to six weeks post-injury, and recruiting participants after discharge.

### Sample

Despite these efforts, only 108 patients, 35% of the target, were recruited. This reduced the statistical power of the study and contributed to equivocal results for most outcomes except MPC.

Approximately 80% of participants were followed up, with most contact by phone, given rural remote residence and/or COVID-19 restrictions. Reasons for loss to follow-up included death and illness; unsuitable timing resulting from ‘sorry time’ (bereavements), travel, reliance on busy relatives; and lack of contact details.

in our region of highly transient populations... that made [follow-up] a little bit challenging. (Regional RSC)

Although follow-up was high and blinding of Assessors was maintained, data from those lost to follow-up could not contribute to findings. COVID-19 likely had a confounding effect in favour of the controls.

### Data collection

Data collection from all trial participants faced several operational challenges. Key evaluation sub-themes related to standardised assessment tools, MPC data extraction, service data quality, ABIC activity data completeness, CST data, and participant perspective, are outlined below.

Assessment tools. In the absence of assessment tools validated for the Aboriginal context, Assessors administered baseline and follow-up tools standardised on the general population. The RCT approach required validated, relatively inflexible assessment tools that placed demands on Assessor skills and patient participation, likely affecting the cultural security of the assessment process as well as compromising the quality of the data. The number and complexity of tools, many of which asked sensitive questions, caused fatigue and incomplete data.

A combination of other factors made data collection challenging:

many of the participants had communication impairments from their brain injuries… some … had language barriers in terms of speaking different types of Aboriginal English (Metro, Blinded Assessor)

Assessors used ‘clinical yarning’ methods [42] and Aboriginal English terms where possible. We do not know the extent or effect of word changes, but they too may have affected the validity and reliability of patient data.

When I was doing the HADS scores, the descriptor ‘feeling of butterflies in my stomach’ had no meaning for some patients; so I had to change it to ‘a little bird flapping in your tummy’. (Metro, Baseline Assessor)

sometimes the next of kin or the carer might reword something I'd said (Metro, Blinded Assessor)

COVID-19 restrictions limited access to patients and most assessments were completed by phone or telehealth. Some Assessors found this convenient:

Telehealth ones actually felt quite fine in terms of length because I knew people were at home and comfortable. (Regional, Baseline Assessor)

Others commented on limitations in information gathering:

the [patient] questionnaire where you ask them how was their experience in hospital, face-to-face they tend to tell you an awful lot more, whereas over the phone they just go, oh yeah, it was ok. Or oh, you know one nurse was annoying. But face to face they'll talk to you much easier. (Metro, Blinded Assessor)

The persistence and flexibility of Assessors, indirect help from ABICs, clinical networks and building relationships with patients during baseline assessment, enhanced follow-up. Although 95% of baseline assessments were fully completed and 80% of patients could be followed up, few follow-up assessments could be fully completed: 17% at 12 weeks, 11% at 26 weeks.

All Assessors were non-Aboriginal women, which might also have had some impact on data collection:

in a couple of cases they felt a little confronted … with the assessors who were all white like myself and not Aboriginal. If … those assessors could have been Aboriginal, …it would have been hugely helpful. (Metro, RSC)

MPC data. Extraction of key data from clinical records was achieved to the standard required to test the secondary outcome, MPC, using an innovative data extraction tool developed by the project team.

Service data. With no consistent state-wide electronic allied health data collection process, diverse individuals (unpaid champions and paid employees) collected data using different hospital systems. Occasions of Service (OoS) data are therefore unlikely to be complete or comprehensive, albeit of similar quality for control and intervention periods.

Patient participant experience. A patient experience survey, developed specifically for the study, was administered at 12- and 26-weeks by the blinded assessors to both control and intervention patients. This survey was culturally appropriate, but brief. Attempts to obtain patient participant views on the ABIC service after completion of the intervention period were largely unsuccessful predominantly due to difficulties in connecting with patients and likely exacerbated by the need to use new interviewers (who were independent for research purposes).

ABIC activity. Data quality varied. Patient visits were carefully recorded, but recording of specific activities varied with difficulty in categorising activities and prior ABIC experience with databases The Data Manager provided support for data collection, including an option to record on paper.

I personally had a lot of phone calls and emails to tech lady over there… you can't like learn it over books or something, you got to do it yourself. To pick out what's going on (Regional, ABIC)

CST evaluation. Data were collected from attendees following face-to-face sessions and online modules. Completion of questionnaires from face-to-face sessions was > 80%. Only 50% of participants completed surveys relating to the online content due to time constraints for staff to complete these modules and an unsatisfactory initial online platform.

#### Project management (Fig. 2, lower RHS)

The process evaluation data indicates that the project was managed with attention to detail, professionalism, flexibility, cultural responsiveness, and compassion. Project management team staff developed strong, supportive relationships with HRW trial staff (ABICs, RSCs and Assessors). They maintained communication with and sustained the engagement of multiple partners, stakeholders, and on-the-ground hospital staff throughout the 5-year study. The time and effort required to ensure the rigour of the trial, cultural security in all processes and stakeholder engagement, as well as to meet bureaucratic requirements did, however, place significant demands on the small project team. In this section, the project management sub-themes are merged into ‘Trial staff recruitment, training and support’, and ‘Managing complex demands.’

### Trial staff recruitment, training and support

HRW recruited suitable research staff for the different roles, using existing networks. Notwithstanding effort to recruit demographically diverse staff, all RSCs and Assessors were non-Aboriginal, so more cultural training and support was required. The content of training and level of support varied by HRW role, although not tailored to the specific knowledge of every participant.

cultural security training that I was able to attend was one of the best I've ever attended. (Regional, RSC)

Not all HRW staff, especially in regional sites, had intervention research experience or skills, and Assessors needed additional, time-consuming, specialist training on the assessment tools. HRW research training was well received.

This is the first kind of research I've been involved in, because I’m new to the role. And they were always very happy to help and answer any questions that I had, and that made a big difference. (Metro, RSC)

Interviews with Assessors, RSCs and ABICs consistently reflected unreserved satisfaction with the responsive support from project team members.

there were a few clients that came in that I don't know if they're eligible or not, really complex situations, and they were very always available for me to call and talk it through to discuss whether or not they would be eligible. (Regional, RSC)

Principal investigators at the hospital sites were generally very supportive, facilitating trial processes.

at least three of us had the same principal investigator and she was very helpful and happy to support and assist. She did, yeah, really great support from her. (Regional, RSC)

### Managing complex demands

The demands of project management required flexibility, hands-on interaction, skill, and cross-cultural understanding. Partnerships with Aboriginal organisations throughout the State created a network considered a strength of HRW. The numerous and varied organisational partnerships required navigation of diverse governance and administrative systems. Multiple ethics committee approvals were needed. Close contact was maintained with all administrative bodies regarding amendments required in relation to COVID-19 and legal next-of-kin changes. These requirements compounded the small project team’s workload.

Due to the stepped-wedge study design and multiple employing organisations with differing administrative processes, implementation of ABIC positions at the various partner locations required considerable negotiation, travel, and time to organise as staffing changed over the intervention period. Senior staff-turnover at some organisations required original agreements being re-visited and/or re-negotiated. Different levels of ‘site-readiness’ needed ongoing communication and support for hospital sites, ABIC employers, and HRW staff including ABICs. Given the time delay between study initiation and start of the intervention at each site, ongoing communication with research sites employers was also needed to remind them of the study, the ABIC role and their time/space commitments and navigation of cultural safety in the workplace. Extensive support from the project management team enabled ABICs to work from home with a laptop and online support during COVID-19.

Communication with the diverse partners was resource intensive, maintained through regular meetings at different levels of the partner organisations, site visits, HRW newsletters and ultimately, presentation of results. Overall, management and logistical planning for HRW was under-resourced, with demands increasing over time, somewhat offset with fewer participants being recruited than anticipated. The management team contributed considerable non-funded, out-of-hours activity to respond to emerging demands:

I often find my role is helping ABICs break down barriers so that they can do their job…there’s a lot of supporting going on and a chunk of that’s out of hours as well. (Project Co-ordinator).

Table 6 summarises the process evaluation criteria for each aspect of trial process implementation (Research design and processes, and Project management); provides answers to the question ‘Implemented as planned’ (Yes, Partial, or a statement of how implementation failed to meet the criterion); with comments, including potential effects on HRW project outcomes.

**Table 6:** Evaluation of trial processes against implementation criteria

Component	Implemented as Planned?	Comment
**RESEARCH DESIGN AND PROCESSES**		
Study Design Stepped-Wedge RCT	Successfully sequentially introduced interventions Step 1 extended	COVID-19 potentially violated the time-related assumption of design Extension due to slow recruitment
Participant recruitment and sample size	Substantial under-recruitment (35% of target) Changes to protocol: * verbal consent * community enrolment * recruitment window extended	Considerable effort put in, however, COVID-19, legislation changes, and limited timely identification of potential participants, difficulties identifying TBI patients exacerbated relatively small pool of potential participants
Follow-up of participants	Relatively high follow-up rate (~ 80%)	Similar in control and intervention groups
Blinded Assessors for follow-up	Yes	Caution that they remain blind to exposure status
Data collection		
- Assessments	Partial Low rates of fully completed assessment data at 12 and 26 weeks	Tools not always culturally appropriate Number and complexity of tools reduced data quality and completion; similar effect on intervention and control groups Assessors all non-Aboriginal women
- Minimum processes of care	Yes	Innovative development of tool Good quality data collected; resource intensive
- Service data	Partial	Alternative sources had to be used, diverse data collectors, quality uncertain but similar between intervention and control groups
- ABIC activity data	Partial	Varying completeness and quality between ABICs
- CST data	Yes	Good data from F2F face-to-face; online responses limited but good quality
- Participant perspectives	Partial	Patient experience survey relatively good quality, but brief Participant interviews not completed
**PROJECT MANAGEMENT**		
Recruitment of staff	Yes	Appropriately skilled staff; Assessors all non-Aboriginal
Training and support of staff	Yes	Extra training provided; Responsive to needs of staff
Overall management of project	Yes	Excellent project management; responsive, flexible, strong problem solving & communication; organized
Cultural security of the overall project	Yes, recognising outer and inner setting not under control of HRW project	Co-design of HRW by Aboriginal and non-Aboriginal investigator team; Co-design and co-facilitation of CST; Management dealt with CS issues as they arose in workplaces; See also ABIC section
Management of ethics and governance	Yes	Excellent attention to detail obtaining approvals, additional amendments and keeping abreast of outer setting changes
Budget	Yes	Underbudgeting of many components meant substantial additional time demands on Management staff
Maintaining partnerships	Yes	Crucial regular communication

### Implementation: Aboriginal brain injury coordinator (Fig. 2, upper LHS)

We begin process evaluation of the interventions with review of the ABIC role (Table 2). Relevant contextual and individual factors are considered before considering the intervention in the context of these factors.

#### Contextual factors

State-wide workforce shortages, particularly rurally, reduced the available recruitment pool for ABICs. Assisted by local networks, HRW successfully appointed Aboriginal people to the ABIC role reflecting feasibility of recruitment to the role. (There were no adverse events during the trial, further suggesting that wider implementation of the ABIC role in health systems is feasible.)

Models of employment varied across regions and employers. One AMS added the ABIC role to an existing National Disability Insurance Scheme [43] role. Multiple data sources reinforced the importance of the role of a coordinator to support Aboriginal brain injury sufferers and their families/carers being filled by an Aboriginal person.

Mob working with mob. (Metro, ABIC).

Some resignations occurred for personal reasons but also due to small participant numbers in some regional areas. When an ABIC position was vacant (26% of the total intervention period), another ABIC covered the vacancy. Gaps were more evident in regional sites, with less availability of potential ABICs, contract delays and COVID-19 all contributing.

#### Individual factors

Although ABICs were mostly living in the allocated region, some were originally from elsewhere and, due to the diversity of people across and within regions, the ABICs were often working cross-culturally themselves.

The ABICs were all female and suitably qualified, many having years of relevant experience in senior Aboriginal health worker or nursing roles. The ABICs were generally selected on, and proved to have, self-confidence to perform diverse tasks including liaison with hospital staff, advocacy, contacting external agencies, and giving presentations. Computer and data entry skills varied. Although cultural preference for same-sex health workers is often cited [44], no ABIC commented on this presenting any specific issues.

#### Implementation of the ABIC intervention: interaction between individuals and context

Operationally, ABICs faced a number of challenges. Mostly, these challenges are related to the interaction between individuals (ABICs, patients/families/carers, health service staff, project team members) and contextual factors outside the control of HRW. Key themes include the need for role clarity and boundary definition, training and support, ABIC activities, and the contribution of ABICs to cultural security.

### Role clarity

Role clarity was challenging in hospital and work settings due to ABIC being a novel role, the potential for overlap with Aboriginal Health Liaison Officers (ALO) (who organise logistics in hospital), delineation of the ABIC role of advocate/support versus direct clinical service, and tensions between having research versus clinical responsibilities in some host organisations.

We had to do a lot of following up with social workers, ALOs, all that kind of stuff… because it’s a new role, a lot of people weren’t too sure… that made it tricky at times… having to repeat our role, and that we’re part of research. (Metro, ABIC)

Role boundaries were also stretched when families and staff requested ABICs get involved in situations involving policy, courts, guardianship, and health conditions other than brain injury. ABICs were sometimes seen as being responsible for raising workplace cultural awareness.

A lot of guardianship issues, a lot of hearings, things like that. That was quite tricky. And having to speak to family about that, and even family not knowing the processes … we learned a lot as we went along. (Metro, ABIC)

### Training and support

The project team and local employers helped clarify roles and provided ABICs with further input about brain function, rehabilitation, and use of REDCap. Initial 12-h ABIC training was supplemented by regular clinical supervision sessions with HRW brain injury staff and a Neurocare Nurse; this increased during the trial from monthly to fortnightly sessions. Workplace support varied. Cultural support and mentoring occurred spontaneously within the ABIC team. External mentoring from an Aboriginal health professional was implemented later in the study.

I reckon if we would have had like hands on training with it... showed us a bit. How to do it a bit quicker. Yeah, because it was new. I never seen this program before in my life…we would have benefited from extra training or something. (Regional, ABIC)

On-the-job support for ABICs was also provided by the HRW project management team. Regular supervision/mentoring meetings were reinforced by strong relationships with the project co-ordinator. Project team members acted as advocates for the ABICs at their workplace as needed.

The drive is to support them to do what we said in the protocol… then there’s the other reality of letting people have the space to grow the role in a way that is inherently culturally informed. (Project Co-ordinator)

The ABICs all commented on the importance of peer support in their role:

other ABICs you can sort of, you know, you run things by each other and have a bit of a chat about the differences in each community and that sort of thing. (Regional, ABIC)

### ABIC activities

ABIC follow-up of patient participants for post-discharge support was complicated by the same difficulties facing Assessors, including cultural commitments and changes in patient participant contact details. This affected compliance with the minimum number of ABIC visits stipulated in the study protocol, ranging from 1–19 across the six-month period. A third (31%) of visits were face-to-face, with the remainder occurring via telephone. Some patient participants did not want or need all planned six visits. The part-time nature of the ABIC role, hospital location (regional rather than metro), and smaller/already heavily committed regional referral and communication networks limited ABICs’ flexibility for follow-up.

In regions where the ABIC workload was low, the ABICs working for health service employers were pulled into other roles prioritised by their employers.

The only problem for me it was like it was short of staff. So I had to, like I know been allocated one day to do this. But sometimes it takes more than that more than a day like, you know. We're short of staff here. (Regional, ABIC)

### Cultural security

Receptiveness to the ABIC role varied across hospital sites and workplaces. In some workplaces with little history of employing Aboriginal staff, tensions were experienced related to isolation and lack of appreciation of personal/family/cultural commitments as well as conflicting role expectations.

it was a lot of cultural awareness stuff that probably needed more doing. Yeah. Even if there would have been - definitely wrong acknowledgement of country, or the colours or the flags, you know, anything. Would have made me feel a little bit more comfortable coming into this position where it's just all wadjellas (non-Aboriginal people) and maybe one or two other cultures here. Yeah, cause it was like, like a fish out of water, you know, foreign territory. (ABIC)

Hosting of two ABICs in one non-Aboriginal organisation provided significant benefits in terms of the collegiality and support available.

The ABICs’ flexibility, responsiveness and cultural appropriateness were notable features of HRW:

on a few occasions they came [on] the day of the transfer to another hospital … They [ABICs] were always lovely for me to deal with and got back to me quickly and were flexible with their times. (Metro, RSC)

what's also as important, you know, like our mob not understanding medical terms as well sometimes, like with the discharge summary, things that can get quite complex. And I think having the ability to break that down and simple terminology is a really big thing as well. (Metro, ABIC)

#### How effective was the ABIC role?

While statistical results found no significant impact of the intervention on patient quality of life, interviews with hospital staff, HRW project team and supervisors in ABIC work settings and case studies were all overwhelmingly positive about the ABICs and the support they provided. Although self-reporting of their activities was unreliable, observation and monitoring in diverse situations affirmed this assessment. ABIC interviews revealed numerous instances of support for patients and families navigating medical (and sometime legal) processes as well as providing support to patients isolated from family and Country. Increasing hospital and health staff awareness of participants’ cultural, social, and emotional needs, including family and community context and general advocacy, was reported. The ABICs’ role in the community post-discharge was especially valuable, particularly maintaining contact with patients and their families, demonstrating that ‘someone cared’, emphasising the importance of rehabilitation appointments, making links with local services, and ensuring that participants knew about service entitlements.

I think it's really, really helpful for patients to have somebody in the community, even better that it's an Aboriginal coordinator, so it's someone that they're more likely to be comfortable to talk to, to help them connect with those services, because I think that's a huge issue and a huge reason why we have such a disparity in the connection with services between Aboriginal and non-Aboriginal patients. (Regional, RSC)

Table 7 summarises the results presented in this section, addressing the question, ‘To what extent was the ABIC service implemented as planned?’ Further details of adaptations to the ABIC service are provided in Supplementary Table 4.

**Table 7:** Evaluation of the ABIC service against planned criteria

Criterion	Delivered as Planned	Comment
Site coverage	Partial	All sites covered, but not all the time, particularly regional sites. Average coverage was 74% (range: 39–100%). ABICs from other regions covered vacancies
Place of employment	Partial	Varying host workplaces, with different work conditions/arrangements
Recruitment criteria	Yes	All Aboriginal with appropriate experience; all women
Training and support	Yes—additional required, requested and provided	Well supported; Team responsive to additional training/ support needs
Prescribed # scheduled visits/contact	Partial (59% received minimum 6 visits/contact) ‘Visits’ included telephone interactions	All received visits; Not all received scheduled 6: some more, some less. Some participants did not want or need more ABIC visits/contact
Modes of contact	Yes, although less face to face than anticipated (31%)	COVID-19 impact large; phone contact was generally shorter
ABIC activities	Assumed yes, but recording of patient-related and other activities suboptimal	Based on interviews with all staff, and type of training/support requested, however ABIC activity data not reliably collected
Adverse events	Yes (no events)	Close monitoring and all RCT requirements met
Cultural security of ABIC intervention	Yes, recognising that outer and inner settings were not under the control of HRW project	ABIC peer/team support; Co-design of ABIC training; Cultural mentor for ABICs; ABIC interviews; Management dealt with cultural security issues as they arose in hospital and ABIC work settings; Public recognition of HRW and ABIC in awards, including Aboriginal sector

### Implementation: Cultural Security Training (CST) (Fig. 2, upper RHS).

#### Contextual factors

Outer and inner setting factors that proved challenging to implementation of the CST included geographically dispersed sites, varying site readiness, site-specific differences in local culture, and competing demands on attendee time. Travel and hospital access restrictions during COVID-19 added to these complexities.

#### Implementation of the CST intervention

With some necessary changes made within the intervention design to meet contextual challenges, the CST intervention was delivered largely as planned. Aboriginal and non-Aboriginal co-facilitation, development and delivery of the specified content, the number and cultural diversity of hospital staff attendees across the sites, and attendee completion of the face-to-face component all met the evaluation criteria. However, online component completion rates were low and the plan to deliver CST every 6 months to each site that had entered the intervention period could not be achieved. Participant remarks in interviews and trainee evaluation surveys confirmed the value of the training and provided insights into how it could be improved. In this section, we review the CST intervention by theme. A summary table, Table 8, is provided below, with further details of adaptations to the CST provided in Supplementary Table 4.

**Table 8:** Evaluation of the CST against planned criteria

Criterion	Delivered as Planned	Comment
Co-facilitation of workshops	Yes	Aboriginal co-lead of CST implementation HRW clinician plus a suitable Aboriginal presenter always co-presented; local Aboriginal co-facilitator 95% of time. Aboriginal facilitators effective and appreciated by attendees and HRW staff
Delivery of specified content	Yes	Cultural security, brain injury and minimum process of care all covered as planned. Content tailored to local Aboriginal cultural and trial site contexts, deemed useful by attendees. Crowded content at times meant running overtime; time restrictions curtailed discussion
Three hours of face-to-face sessions	Yes	Some sites preferred three one-hour CST sessions
At least 20 hospital staff attend CST across multiple time periods at each site	Yes	250 people attended 18 face-to-face workshops
Each site received CST every 6 months during the intervention period	No	Workshops took place at all sites COVID-19 restrictions, site preferences & HRW staffing resources affected scheduling decisions, including prioritisation of sites in later steps
Diverse attendees (experience and role)	Yes	Medical and management staff under-represented. Aboriginal hospital staff present at all workshops
Completion of face-to-face and online components	Partial	Fewer than 5 attendees failed to complete the full three-hour face-to-face training Despite major effort by the research team, only 50% of face-to-face participants completed online component, limited by IT platforms and competing demands on hospital staff time

### Aboriginal facilitation and content creation

Consultation with local Aboriginal and non-Aboriginal staff at each site was crucial when tailoring workshop content to be locally relevant. CST was developed and delivered with co-leadership and co-facilitation by Aboriginal and non-Aboriginal experts, despite challenges in identifying suitable local Aboriginal cultural experts to deliver the CST across the intervention period at each site. The HRW project team put considerable effort into building relationships with co-facilitators and improving skills where needed to ensure consistent and engaging workshop delivery. This resulted in many successful partnerships, which were a key positive aspect of the intervention.

it's a big thing to present, to hold the room, to facilitate. Not everyone has those skills, …or the right, the permission, the whatever, to be talking about Aboriginal culture (Project Co-ordinator)

She [Aboriginal co-facilitator] had people come up to her after and comment, and it was really good to have their ALOs involved and, the ALOs and their value - they felt valued. (Project Co-ordinator)

### CST content delivery

All planned content – cultural security, brain injury, and MSC – was delivered. CST evaluation surveys indicated an overwhelmingly positive response to the face-to-face workshops (mean satisfaction score > 90%). The online component of training was also highly rated, despite a low completion rate.

Attendee CST evaluations point to the value of Aboriginal and non-Aboriginal collaboration in developing and delivering the CST intervention:

The course is exceptionally helpful to guide reflection on interactions with Aboriginal people and how to approach from an Aboriginal perspective. There is a general desire amongst health professionals to treat in a culturally aware manner, now we have the knowledge and tools to do so. Thank you. (Regional, profession unknown)

Loved the discussions and experiences shared by other Aboriginal women/Aboriginal Liaison Officers in the audience. Added further perceptions (Metro, Speech Pathologist)

The diversity of attendees’ experience was carefully considered prior to each workshop to enable facilitators to show sensitivity during workshop discussion. This enriched workshops, but challenged facilitators to deliver all content within the available time. This was particularly evident for the brain injury content, which was delivered by case study and video and was much valued by trainees.

Not all attendees considered their existing cultural security practices and efforts were acknowledged in the sessions:

Truthfully, I found that the advice to listen, social yarn and build rapport with patients, involve family and discharge plan is something I consistently do with all patients, not just Aboriginal patients and so I would have liked a real focus on what else I need to be considering above that. (Metro, Medical)

Both attendees and facilitators felt more time could be spent on practical activities such as role plays.

Would love more opportunity to practise clinical yarning and different ways to approach this (Metro, Physiotherapist)

Some attendees suggested the training was too generic and required more clinical content:

It would have been beneficial to have more practical strategies specific to allied health interventions. For example, ‘story telling’ analogies for cognitive, perceptual, visual deficits etc to support understanding, as well as resources to support education with families. (Metro, Occupational Therapist)

Others requested more training, recommending regular updates for key staff including managers.

### Workshop schedule

Face-to-face workshops – 18 in all – were held at all sites. All face-to-face workshops consisted of three hours’ training, although at some sites they were delivered in three one-hour workshops. Timing of CST roll out at each site was predetermined by HRW’s stepped-wedge design with sites having the option to hold training every six months once they were in the intervention phase of the study. Factors impacting workshop scheduling included availability of trainers, competition with mandatory training and rostering requirements, senior and administrative staff turnover, and seasonal emergency requirements.

Commitment from a logistical and cultural perspective also varied, along with site-readiness for the CST. COVID-19 restrictions on travel and hospital entry added further complexity and it was not possible to deliver as many repeat workshops as expected. Even when workshops could go ahead, social distancing requirements affected the number of attendees. Scheduling of repeat training was prioritised at sites yet to achieve the required number of staff trained.

### CST attendees

Two hundred and fifty hospital staff attended the CST. As planned, attendees had different roles (medical, nursing, allied health, management) and levels of experience. Clinical experience working with brain injury and/or Aboriginal patients also varied. Aboriginal hospital staff were present at all workshops. Medical and management staff were under-represented.

Practices for selection of potential CST attendees differed across sites, with some nominated by hospital administrators and others self-nominating. Because attendance was non-mandatory, self-selection of staff already interested/motivated in cultural security may have biased attendee evaluations and observations at all sites. Differences in individual prioritisation of CST training was influenced by work pressures, competing training requirements, personal enthusiasm for training, and previous experience working cross-culturally.

I mean people thought it was important, but in terms of getting onto the ground level, people saying, when would you do it? Never a good time to do it… we haven't even got time to do our compulsory training. (Chief Investigator)

Systems for organising staff attendance were complex, however often facilitated by hospital staff CST ‘champions’ identified by the HRW project team.

it was probably one of the hardest parts of the study because we needed a lot of people to give approvals and then to get the word out and get people signed up... they do have to be rostered on, or if they're rostered on and doing training, they have to backfill them so there is a significant logistical thing there. (Project Co-ordinator).

### Completion of CST components

Almost all CST attendees completed the face-to-face component (fewer than 5 of the 250 did not complete). However, completion of the online component was sub-optimal (50%). This was initially due to unanticipated technical issues, e.g., ongoing technical support, Health Department firewall issues, and individual access. Completion was also impacted by the need for trainees to complete the modules in their own time, within the context of the additional personal and work demands of COVID-19, the competing demands, motivations, and experience that influenced them to enrol for the training in the first place, and potentially online component design.

the online modules had too many scenarios.(Metro, Nursing)

too extensive and repetitive, ... there was no opportunity for me to complete during work hours. A shorter more succinct course might be more beneficial. (Metro, Physiotherapist)

Efforts to increase online completion included increasing the post-workshop online access period, regular reminders, and certificates of completion. Piloting the CST prior to implementation would have identified some of the technical challenges and helped to identify specific aspects of the online content and completion requirements that might be modified to improve the completion rate.

## Discussion

This process evaluation found that the complex HRW trial was implemented to a satisfactory level despite exceptionally challenging circumstances related to outer and inner setting factors, including COVID-19. The shortfall in patient participant recruitment was recognised but could not be overcome. Crucially, the vulnerability of the stepped-wedge study design to time effects – in particular, related to COVID-19 – likely had a major bearing on recruitment and the neutral trial results. Participant follow-up was high relative to current levels within the health system, although losses to follow up further reduced data points for analysis. Despite the highly committed Assessor team, the lack of culturally appropriate assessment tools and no Aboriginal people as Assessors likely influenced the quality of assessment data. Recruitment, training, implementation, and appropriate supervision of the ABICs was shown to be feasible and, within the confines of the data collected, well-received. The CST involved complex logistics, but nevertheless incorporated the content, style and Aboriginal co-facilitation as intended; all rated highly although online components were often incomplete, and some sites did not receive training in each step, diminishing the intended intensity of the intervention. Trial processes and interventions would have benefitted from more pre-trial piloting. Project management was responsive to rural and metropolitan stakeholders, staff, participants, inner and outer setting factors, with adherence to RCT requirements and documentation. Cultural security of the project was always prioritised by the Investigator and Management teams, with Aboriginal leadership, partners and network providing crucial cultural and logistical support.

Despite the equivocal results of the primary and most secondary outcomes, significantly greater implementation of the MPCs was observed for patients seen during the intervention vs control periods. Eleven MPCs (for example, timely allied health assessments received, Aboriginal Liaison Officer contribution, use of interpreters, discharge plans developed with family, communication between rural and metropolitan hospitals) [10] were based on clinical guidelines and best practice statements, and can be considered system-level indicators of quality of care. MPC were explicitly covered in the CST workshops, suggesting an impact related to training, although other contextual factors may also have influenced MPCs. Overall satisfaction with hospital experiences during the intervention vs the control period was shown in surveys administered to HRW patient participants, with comments reflecting a more active Aboriginal presence. Both the CST and the ABIC interventions would have influenced these positive results which suggest that these interventions show sufficient promise to warrant further exploration and implementation on a broader scale.

HRW was a pragmatic trial as reflected by its real-world setting, real-world population, relevant outcomes, and comparator group. The advantage of conducting an RCT was the academic recognition such studies receive. Nevertheless, strict adherence to a rigid protocol and the need for precise and comprehensive measurement and blinding, while adding to the rigour, potentially compromised cultural security for participants and was logistically challenging. The research was conducted in the intercultural space, where there is ongoing tension between Western and Indigenous ways of knowing being and doing [45–47]. The process evaluation identified this tension between fidelity to protocol versus the need for adaptability and flexibility for optimal implementation. Few Aboriginal-specific randomised controlled trials have shown intervention effects, often due to the complex interventions introduced, challenging environments, and small numbers recruited [48–56]. Other well-conducted, real-world intervention studies using mixed methods might be a preferred design to ensure optimal benefit, meaningful outcomes and more appropriate tools, and learnings [53, 57, 58]. Additionally, research funders need to invest in mixed-methods studies which co-design, develop and pilot interventions with Aboriginal Australians, including process evaluations to refine approaches.

The process evaluation highlighted how intervention research, such as HRW, can be successfully undertaken in the Aboriginal brain injury context on a large scale and with cultural security and Aboriginal contribution at its core, despite the tyranny of distance and multiple contextual and health system barriers. The inclusion of both rural and metropolitan sites provided whole-of-system analysis, with challenges for rural sites and patients uniquely described for this clinical group. The evaluation identified that good training, appropriate mentoring, a strong Aboriginal role at the interface with patients and in service planning, and statewide, centrally co-ordinated services with strong local presence embedded in the inner setting, are the essence of overcoming health system challenges.

HRW strove for best practice in RCTs for complex interventions as recommended by the Medical Research Council [22]. Until recently, most process evaluations comprised almost exclusively of retrospective interviews [59–62]. Much of HRW’s process evaluation was prospectively and concurrently undertaken alongside HRW, providing detailed insights into contextual factors, dynamics, actions, and learnings, using varied and rich sources of data. Learning was fed back to the management team as the trial unfolded, supporting quality improvement of processes within the RCT and helping with the interpretation of statistical findings of the trial.

Besides feeding into the implementation of the interventions, the evaluation also provides crucial information to inform designs of future health service delivery for this population. Our findings support the increasing use of culturally secure navigators in health and disability care, with some Australian States now considering this approach foundational in support of people with disability [63]. The learnings from implementation of the intervention will influence how these roles are implemented. This includes recommendations regarding approaches to follow-up, in-reach model for Aboriginal health navigators, types and mode of training/support needed, and how to operationalise person-centered care and cultural security in both rural and metropolitan services. Analysis of HRW research processes as well as implementation of the actual interventions formed a strong component of the evaluation and contributes guidance for future research in the areas of stroke and TBI rehabilitation which have only been sparsely explored, with no process evaluation of rehabilitation specifically focused on Aboriginal brain injury survivors available to date. Indeed, the process evaluation provided data for conference, stakeholder and media presentations as HRW was being rolled out, enhancing potential for translation of learnings after completion [64].

The evaluation team included Aboriginal and non-Aboriginal researchers, with effort made to ensure cultural security of the evaluation process. This included privileging the perspectives of the senior Aboriginal team members in the design, data collection and analysis of the results. Inclusion of Aboriginal people, including one stroke survivor, in data collection built some capacity, while highlighting the need to further develop Aboriginal evaluators for the future. An important shortcoming of the evaluation was the limited evaluation data collected from the Aboriginal stroke and TBI participants themselves, particularly regarding their experience of the ABIC. Because of blinding, the short questionnaire administered twice to both control and intervention participants is the only source of information that addressed their overall experience, without direct reference to ABICs. In addition, assessment tools for the HRW outcomes were not always appropriate for the Aboriginal context. The limited resourcing for the process evaluation meant that some members of the HRW investigator team also contributed to the evaluation (‘insider roles’) with complete independence of the evaluation not feasible. However, the funding that was available enabled employment of individuals responsible for sub-analyses that contributed to the overall synthesis of evaluation data. This overlap in roles was also a strength, in that one researcher, embedded within the management team, was able to regularly update process data, ensuring accurate and rich sources of documentation for the evaluation. The use of the CFIR facilitated translation into practice through its implementation focus.

## Conclusion

Despite the hypothesised effect of the HRW intervention on patient quality of life not being achieved, HRW has illustrated the feasibility of strategies to improve cultural safety for Aboriginal brain injury survivors, in particular the ABIC role working with mainstream hospitals and increased clinical content in CST. It has built capacity for intervention research in regional settings and provided evidence of direct and indirect benefits to patients. The process evaluation has been a vital part of the RCT undertaking, providing contextual analysis of the myriad of factors affecting all aspects of the trial. The strategies developed and lessons learned as outlined in this evaluation lay the foundation for future intervention research and implementation designs to build robust models of culturally secure rehabilitation for Aboriginal brain injury patients.

### Supplementary Information

Supplementary Material 1.

Supplementary Material 2.

Supplementary Material 3.

## Data Availability

Neither of the datasets from the current study or the parent study (HRW) are available as per ethics approvals and Indigenous data sovereignty requirements.

## Abbreviations

ABI: Acquired Brain Injury
ABIC: Aboriginal Brain Injury Coordinators
CFIR: Consolidated Framework for Implementation Research
CST: Cultural Security Training
HREC: Human Research Ethics Committee
HRW: Healing Right Way
MPC: Minimum Processes of Care
RCT: Randomised Controlled Trial
RSC: Research Site Contacts
TBI: Traumatic Brain Injury
WA: Western Australia
