# Supplementary material for: Process evaluation of a randomised controlled trial intervention designed to improve rehabilitation services for Aboriginal Australians after brain injury: the Healing Right Way Trial
Source: BMC Health Serv Res. 2024 Aug 20;24:946. doi: 10.1186/s12913-024-11390-5 (PMC11334317; doi:10.1186/s12913-024-11390-5)
Supplement: Supplementary file 1 — Supplementary Material 1. [file 12913_2024_11390_MOESM1_ESM.pdf]

Supplementary File 1

Healing Right Way Process (HRW) Evaluation

INTERVIEW SCHEDULES AND SURVEY QUESTIONNAIRES

CONTENTS

Data collection tool	Respondents	Timing of data collection	Page
1. Face-to-face Cultural Security Training (CST) survey questionnaire	All hospital staff who attended a face-to-face CST workshops	After face-to-face session	2
2. On-line Cultural Security Training (CST) survey questionnaire	All hospital staff who completed the online CST material	Within 6 weeks of the face-to-face workshop	5
3. Patient participant survey	Recruited Aboriginal patients with stroke or traumatic brain injury	12-week and 26-week follow-up interview	9
4. ABIC Interview schedule	Aboriginal Brain Injury Co-ordinator	End of project	11
5. HRW management staff interview schedule	Chief investigator<br>Program manager<br>CST program designer/CST delivery<br>ABIC Program designer/ ABIC training and support<br>Data/Operations Manager	Every 3-6 months throughout the project	12
6. Baseline Assessor interview schedule	Assessor who interviewed patient participants when recruited	End of project	18
7. Blinded Assessor interview schedule	Assessor who interviewed patient participants when at follow-up interviews	End of project	20
8. Research Site Coordinator interview schedule	Research Site Coordinator	End of project	22

1. Face-to-face Cultural Security Training questionnaire

Thank you for supporting this research by attending the face-to-face training session(s) run at your workplace. Your involvement and engagement in this professional development training is most appreciated. Please take a few minutes to complete the questions below.

General Information:

Your professional discipline			Your gender		M	F
Your level of professional experience	< 1 year	1-5 yrs	5-10 yrs	10-20 yrs	20-30 yrs	>30 yrs
Years of brain injury (stroke/TBI) experience	< 1 year	1-5 yrs	5-10 yrs	10-20 yrs	20-30 yrs	>30 yrs
Name of your workplace					Date of training	
Names of your presenters						

Please indicate how this training was presented for you:

As a single 3 hour session?			
As three 1 hour sessions? (Please tick those you attended)	Session 1	Session 2	Session 3

Please tick the box that best reflects your view of the specific aspects covered in this course:

	**Very useful**	**Quite useful**	**Neutral**	**Not very useful**	**Not at all useful**	**Other** (see below – space for comments)
Knowledge of local cultural issues delivered by a local Aboriginal cultural security trainer						
Explanations of *cultural security* and how they apply to my workplace						
Explanations of *clinical yarning* and how this can be applied in my workplace						
The use of case scenarios of Aboriginal people with acquired brain injury to support learning						
	Very useful	Quite useful	Neutral	Not very useful	Not at all useful	Other (see below – space for comments)
The opportunity to consider the cognitive, behavioural, and communication impairments which commonly occur after stroke or traumatic brain injury						
The focus on practical strategies, good communication skills, and culturally secure *relationships* with Aboriginal patients and families						
Experiential learning and the opportunity to reflect on working with Aboriginal patients and families						
Having the time to *team-build* with colleagues						
Having the time to consider the policies or ***reconciliation action plan*** for my workplace						
An opportunity to develop best practice principles in my workplace						

Please tick the box that best reflects your view of the impact of this course:

	**A lot**	**A little**	**Somewhat**	**Not at all**	**Other** (see below – space for comments)
It has been **useful** in relation to my team's work or individual practice					
It will make a **difference** to the way I work with Aboriginal patients and families.					

If you answered *other* or would like to comment further on any aspects of the course, please do so here:

Thank you!

2. Online Cultural Security Training questionnaire

Thank you for supporting this research by both attending the previous face-to-face training session(s) run at your workplace and doing the online training (link url). Your involvement and engagement in this professional development training is most appreciated. This questionnaire will focus on your feedback on the online modules and your reflections on this professional development overall. Please take a few minutes to complete the questions below.

General Information (complete or circle as appropriate)

Your professional discipline			Your gender		M	F
Your level of professional experience	< 1 year	1-5 yrs	5-10 yrs	10-20 yrs	20-30 yrs	>30 yrs
Years of brain injury (stroke/TBI) experience	< 1 year	1-5 yrs	5-10 yrs	10-20 yrs	20-30 yrs	>30 yrs
Name of your workplace						
Approximate time spent completing online modules	1 hour		2 hours		3 hours	
Having time between the face-to-face and online training	Helpful for reflection and applying the experiences and information to practice		Neutral		Not helpful for reflection and applying the experiences and information to practice	
Access to the site through ECU's Health*Info*Net	Easy access		Difficult to access	Comment:		
Quality of the site developed for this training on ECU's Health*Info*Net	High quality information, presentation and links		Poor quality information, presentation and links	Comment:		
Importance of certificate of completion	Very important		Neutral		Not important	

Please tick the box that best reflects your view of the specific aspects covered in the online modules

	**Very useful**	**Quite useful**	**Neutral**	**Not very useful**	**Not at all useful**	**Other** (see below – space for comments)
**Section on "learning from my patients"**						
The use of case scenarios of Aboriginal people with acquired brain injury to support learning						
	**Very useful**	**Quite useful**	**Neutral**	**Not very useful**	**Not at all useful**	**Other** (see below – space for comments)
Opportunities to view video clips of people's experiences						
Quiz questions to reinforce learning						
**Section on "learning from my colleagues"**						
Having scenarios and video clips of people's experiences						
Practical strategies, and a *framework* for working with Aboriginal patients and families						
Thinking through practices for: admission, assessment, family involvement, collaborative goal planning, ongoing referrals, discharge planning (please comment more specifically below if you wish).						
**Section on "systemic practices"**						
Having the time to consider the policies or ***reconciliation action plan*** for my workplace						
Following suggested **links** to other relevant resources						
An opportunity to develop best practice principles in my workplace						

Please tick the box that best reflects your view of the impact of this course

	**Strongly agree**	**Agree**	**Neutral**	**Disagree**	**Strongly disagree**	**Other** (see below space for comments)
Both the face-to-face and online aspects of this training have been generally **useful** in relation to my team's work or individual practice						
This training has made a **positive difference to my attitude** towards working with Aboriginal patients and families with brain injury.						
This training has made a **positive difference to my skills/knowledge** in relation to working with Aboriginal patients with brain injury and their families.						
The training was helpful in addressing the specific issues for **managing brain injury** for Aboriginal patients and families						
This was helpful in promoting stronger working practices with **Aboriginal Liaison Officers and/interpreters**						
The overall training will make a **difference to the way I communicate/yarn** with Aboriginal patients and families.						
The overall training will make a **difference** to the systems in my workplace for Aboriginal patients and families.						

If you answered *other* or would like to comment further on any aspects of the course, please do so here:

Thank you!

3. Patient participant survey

Enhancing Rehabilitation Services for Aboriginal Australians after Brain Injury

Participant Questionnaire

Hospital Service

Tell us what you think about…

Being in hospital and then coming home

Tick the box you think is right for you…

	Yes – it was good 🙂	In the middle 😐	No – it was not good ☹
Looking back… how I was looked after in hospital			
How staff yarned with me and answered my questions			
How staff talked with my family			
How staff planned for what I wanted when I left hospital			
Receiving the information I needed			
Getting therapy if I needed it			
Preparing me for keeping busy and seeing friends			

Anything else you want to add?

____

____

____

____

____

____

____

Thank you for telling us how it has been for you.

4. Aboriginal Brain Injury Coordinator Interview Schedule

**Topics and related questions for semi-structured interview for the Aboriginal Brain Injury Coordinators**

- Tell us a little about your role in this project.
  - What did you do?
- What did you think about your training?
  - Did you get enough training?
  - What was the most beneficial aspect of your training?/ Comment on the usefulness or relevance of a) orientation b) training c) clinical supervision
  - Is there information/ training you feel would have been useful to receive to help you in your ABIC role? If so, what additional information/ training would you have liked to receive?
- Did the role meet your expectations of what it would be?
- What things, if any, helped you achieve what you wanted to do in your role?
- What things, if any, got in the way of your ability to achieve what you wanted to do in your role?
- Do you feel you were able to help your participants to access rehabilitation services?
  - If so, how did you help them? What did you do that made a difference to them?
  - If not, what were the main things that prevented this?
- What parts of the ABIC service did you feel the participant found the most useful?
- How would you explain your role to someone outside of the project?
  - (Prompt – have a look at the kinds of activities that were originally planned and listed in REDCap – do you feel these captured your activities? If so, which ones would you say you spent the most time on? And if not, why not?)
- What advice would you give to a service that was looking to employ Aboriginal Brain Injury Coordinators?
- What was your experience of being based at the Neurological Council of WA/Aboriginal Medical Service? Can you comment on:
  - your ability to perform your role in a way that reflected the needs of your Aboriginal participants
  - your own feelings of cultural safety in the workplace
  - what you think makes a workplace feel comfortable for Aboriginal staff
- What was your experience of working with the staff at the hospital e.g. medical team, ALOs, allied health team?
- What was your most memorable moment during your work as a Brain Injury Coordinator?
- Do you have anything you'd like to add/share?

5. Healing Right Way Management Team interview schedules

Notes on interview questions

This is a question bank from which the questions will be drawn for each of the guided reflection and interviews conducted. Questions map to different aspects of the Consolidated Framework for Implementation Research. The nature of process evaluation means that some questions may be relevant at specific time points, others may be relevant throughout, and potentially, some may not be relevant given how the program/intervention is being implemented.

Timing for guided reflection and interviews for each key informant can be found as part of the evaluation plan

The 'intervention' includes both the culturally secure training within the hospital setting (including development, delivery and outcomes) and all aspects of the Aboriginal Brain Injury Coordinator (ABIC) role (including recruitment, training, support, and the role itself). When intervention is included in a question, each component of the intervention will be considered separately.

The interview is an opportunity for reflection regarding the implementation plan and progress, hence may result in modification of the plan or intervention.

1. **Questions for the Chief Investigator.**
    - What (if any) complexities have you identified in the last quarter?
    - What (if any) modifications have needed to be made to the intervention? If changes:
        - What modification was made?
        - Why was the change deemed necessary? (e.g. emerging evidence, cost, barriers, stakeholder suggestion...)
        - How has this decision been made?
        - Who was involved in that process?
        - Are there components of the intervention that should not be altered? If so, what are they?
        - To what extent has the intervention been compromised due to things that are happening at each site?
    - Can you describe the plan for implementing the intervention?
        - How detailed is the plan? What is the complexity? Is it realistic/feasible?
        - How are you tracking the progress of implementation based on your plan?
    - What do influential stakeholders think of the intervention and its implementation?
        - What have you needed to do to get the stakeholders on board?
        - Have stakeholders assisted in identifying barriers to the intervention or the implementation?
        - What level of endorsement or support have you received from the leadership?
            - Who are these leaders? Their roles?
            - How do attitudes of different leaders vary?
            - What kind of support have they given you? Can you provide specific examples?

- Has someone (or a team) outside your research team helped you with implementing the intervention?
    - Can you describe this person/group?
    - How did they get involved?
    - What is their role?
    - What kind of activities will they be doing? How helpful will this be? In what way?
- Who is leading the implementation of the intervention at site X? (X- each of the current sites)
    - How did that person come to the role? Appointed? Volunteered? Voluntold?
    - What attributes/qualities does that person have which makes them an effective leader of this implementation?
    - What attributes/qualities does that person lack, and how have you compensated for this?
    - Does the person have sufficient authority to do what is necessary to implement the intervention?
- What is your perception of the preparedness for implementation of the intervention? Eg:
    - Quality of the supporting materials – resources & toolkits
    - Team preparedness
    - Site readiness
    - Are there any unforeseen barriers or facilitators to implementation?
- Do you expect to have (or did you have) sufficient resources to implement and administer the intervention?
    - What resources are you counting on?
    - Are there any other resources that you will need to procure? Where from?
    - What challenges do you expect with respect to procuring additional resources?
- Are you aware of any recent other initiatives or programs that have been implemented in the health service sites or region you are dealing with?
    - What factors helped make it successful/fail?
    - Who were the key players?
    - Can you tell me about how leaders were involved? Who? Their roles? How they helped/hindered?
    - How might these initiatives impact on the patients targeted by your study, and the results of the study?
- What kind of local, state, or national performance measures, policies, regulations, or guidelines are relevant to the intervention?
    - How is the intervention affected?
    - What are the implications for implementing the intervention?
- What aspects of the health system affect the implementation of the intervention?
    - Are there any structural elements of the health system which facilitate/hinder implementation of the intervention?
    - How will you work or how have you worked around structural challenges?
    - What kind of structural or operational changes will be needed to accommodate the intervention? (eg. Changes in policy, information systems, record keeping, other?)

    - What kinds of approval do you need for each site? Who needs to be involved?
- How well does the intervention fit with existing work processes and practices?
    - What are likely issues or complications that may arise?
    - Can you describe how the intervention will be integrated into current processes?
    - How will it interact or conflict with current programs or processes?
- What kinds of other high priority initiatives or activities are happening within the health service sites?
    - What is the priority of getting your intervention implemented relative to those other initiative?
    - Will implementation conflict with these priorities?
    - Will implementation help relieve pressure related to these priorities?
- Can you describe your working relationships with your colleagues?
    - With CI's
    - With project team
    - With other stakeholders (e.g. reference group)
    - How helpful have meetings been?
    - Have you needed to change anything with respect to how you've met, or communicated, with each group?
- Can you describe the activities that have the highest priority for you?
    - For your team?
- In the last few months, who were your 'go-to' people when you needed to get something done or solve a problem?
    - Can you describe recent examples for different types of problems?
- To what extent have you set goals related to the implementation of the intervention?
    - If yes, what are the goals?
    - To whom have they been communicated (internally & externally)?
    - Are changes made based on how things are going? Can you give an example?
- How confident are you that you will be able to successfully implement the intervention?
    - What gives you that level of confidence?
    - How confident are you in your team?
- How would you describe the culture of your team?
    - Do you feel the culture of your team is different from that of the health system? In what ways?
    - How do you think your teams culture will affect the implementation of the intervention?
    - How do you think the culture of the health system will affect the implementation of the intervention? Can you describe an example to highlight this?
- What is the general level of receptivity in the health system to implementing the intervention?
    - Why?
    - Does it differ between sites?

CI questions for when intervention has started:

- To what extent are health service staff aware of the needs and preferences of Aboriginal people with acquired brain injury?
- Have you had feedback from Aboriginal people with acquired brain injury or their families regarding their experiences with the ABIC?
  - What were their perceptions of having ABIC support?
  - Can you describe any specific information you have heard?
  - Can you describe a specific story?
- In general, what level of engagement do you think the health sites are to use the intervention on an ongoing basis? (Stages of Change)
  - Knowledge stage (precontemplation) – knowledge of key aspects of the intervention
  - Persuasion stage (contemplation) – likes the intervention, discusses it with others, buys into it, has positive view
  - Decision stage (preparation) – intends to seek additional information and try it
  - Implementation stage (action) – acquires additional information, uses intervention regularly, and has continued use
  - Confirmation stage (maintenance) – recognises benefits, has integrated the intervention into routines, promotes use to others.
- Who have been the champions of this intervention & its implementation?
  - Were they formally appointed, or was it an informal role?
  - What positions do the champions hold?
  - How do you think they helped with the design of the intervention?
  - How do you think they helped with implementation of the intervention?
  - What kind of behaviours or actions did the champion(s) exhibit? Eg. Getting others on board, problem solving, etc.
- Was the intervention implemented according to the implementation plan?
  - If yes, can you describe this?
  - If no, why not?

2. Questions for the Program Manager

- Do you have a sense of where the site is 'at' with respect to cultural awareness?
  - Are there any other activities going on at the site regarding developing cultural awareness?
  - What is the level of involvement with the project of leadership at the site?
  - Who are the champions for culturally secure practice within the site? What are their roles?
- In terms of delivering the training, what issues (if any) have you come across that are relevant to delivery in other sites?
  - (e.g. site management, ability to release staff for training, 'buy-in' from staff/management)
  - What would be the key pieces of advice you would have regarding organisation and delivery of the training?
- How many different people are you dealing with at each site to implement the CST training?
  - What are their roles?
- What are the challenges/barriers to recruiting participants?
  - Systemic (health, government)?

    - Administrative (e.g. Project related – e.g. protocol)?
    - Site specific?
    - Specific to individuals/personalities and/or families?
- What are the work-arounds/solutions that you've had to come up with for these barriers?
- What are the challenges/barriers to working with the individual sites?
    - Systemic?
    - Administrative?
    - Site specific?
- What have been the changes that have occurred in the last month that impact on the project?
    - Are there any new agencies/organisations are you working with to implement the HRW project?
    - Have key personnel changed?
- What have you learned from implementing the project in individual sites that would be useful for other sites or future projects?
    - Preparation?
    - Implementation?
    - Site requirements (e.g. leadership, key stakeholders, etc.)?
    - Project requirements (e.g. what the HRW team needs)?
- How many different people are you dealing with at each site to implement the HRW project?
    - What are their roles?
- Have you any comments to make regarding:
    - Managing expectations/understanding of HRW in organisations
    - Communication (barriers and facilitators) with organisations
- What have been the challenges/barriers to recruitment to the ABIC role?
- Is there anything else you would like to tell us?

3. Questions for the program designer/CST delivery

- What was the level of engagement of people attending face-to-face training?
    - Were staff present for the entire session?
    - Were there any issues with staff attending on the day(s)?
- What level of adaptation do you believe has been made to the training?
    - Did content differ from what was intended? If so, why?
    - Was the way face-to-face training was delivered different from what was intended? If so, why?
- In terms of delivering the training, what issues (if any) have you come across that are relevant to delivery in other sites?
    - (e.g. site management, ability to release staff for training, 'buy-in' from staff/management, appropriate facilities/equipment)
    - What would be the key pieces of advice you would have regarding organisation and delivery of the training for other sites, or in similar programs?
- Is there anything else you would like to tell us regarding the cultural security training?

- We have focussed a lot on challenges, what were the highlights of the training? What can we learn for the future?

4. **Questions for the program designer/ABIC training and support**
   - Were there any unexpected challenges with delivering the training to the ABIC?
     - Was the training content different to what was intended? If so, how?
     - With hindsight, is there anything that needs to be included in the ABIC training for future sites?
     - What worked well with the training?
   - How do you feel the ABIC was supported by the external stakeholder organisation (NCWA, AMS, etc.)?
     - What was the level of cultural security within the external stakeholder organisation?
     - What were the challenges in managing the relationship with the stakeholders?
     - What worked well for the external stakeholder?
     - What worked well for the ABIC?
     - What are the key considerations for supporting the ABIC in other sites, or in similar programs?
   - Is there anything else you would like to tell us regarding training and/or supporting the ABIC?

5. **Questions for the Data and Operations Manager of the Trial**
   - Have there been any changes to the project from the perspective of your role within the project?
     - If yes, what have been the drivers of this change?
     - How have you needed to change things?
   - Has there been any issues you've noticed regarding the collection and storage of data?
     - If yes, what have been the causes?
     - How have you solved the problems?
   - Have any problems arisen that are related to contract management?
     - If yes, how have these been resolved?
   - Is there anything that has changed with the new sites?
     - What is the reason for this?
     - Will that influence any other component of the study?
   - With respect to your responsibilities within the project, is there anything else important that you think we should be aware of?

6. Baseline Assessor Interview Schedule

ASSESSOR ROLE QUESTIONS

1) Can you please explain your role as a Baseline Assessor for the Healing Right Way study?

2) Please describe any barriers or facilitators you encountered while undertaking the following aspects of your role:

- Recruitment / consent process (e.g. assent, verbal, next of kin, Research Decision Maker)
- Assessment type (e.g. HADS, NIHSS, FIM)
- Assessment mode (i.e. in-person, Telehealth, phone)
- Documentation / hand over of forms (e.g. How accessible were the assessment forms? How easy did you find it to complete and return the forms? How easy did you find it to use REDCap?)

What were the work-arounds/solutions that you came up with? Can you please give some specific examples?

SITE QUESTIONS

3) What level of support did you receive from the Research Site Contact(s)?

- What kind of support did they give you? Can you please provide specific examples?

4) Please describe what else helped you in your role as a Baseline Assessor. (e.g. hospital staff [ALO involvement during assessments], research staff, other people, training/resources, strategies, circumstances)

When applicable (if assessments completed on-site)

5) How would you describe the attitudes of staff at the assessment site and the overall culture of this work environment?

Examples include:

- Assessor requiring access to the patient
- Engagement with assessor as a researcher/non-staff member
- Attitudes expressed towards patients participating in the study
- Attitudes towards the Healing Right Way study

When applicable (if assessments completed off-site)

6) Did you complete any assessments in the community (i.e. outside the hospital environment)?

- Can you please describe the facilitators and barriers you encountered in organising and undertaking these assessments?

ROLE PREPAREDNESS QUESTIONS

7) What is your perception of your preparedness for your role in an RCT surrounding Aboriginal people with brain injury? Please comment on things such as training, resources, and support from Healing Right Way staff.

- How much background information relating to the project did you receive, prior to starting your role?
- How much previous experience did you have working with individuals with acquired brain injury?
- Did you have previous experience working with Aboriginal patients/families?
- Did you feel prepared to work with the Aboriginal patients/families? What helped prepare you?
- Please describe any challenges you experienced working with the Aboriginal patient/families? How did your experiences differ from working with non-Aboriginal patients/families?
- How prepared did the hospital appear for the project in terms of accommodating you as an assessor? (e.g. How prepared did you feel the different wards were for your visit? i.e. did you feel welcome; or have to explain why you were there?)
- How did you find the process of completing all of the required training to be able to execute your role? (e.g. FIM training, using REDCap, cultural security of assessment processes, education relating to the project)
- Do you have any examples of how you used your training?
- What support did you receive from Healing Right Way staff?

8) Please describe any other challenges you experienced in this role. Other aspects of your role may have included:

- Time pressures (e.g. How did you manage having this additional role with your current job?)
- Covid-related issues
- Mode of employment (e.g. getting an ABN, ECU contract)
- Communication with participants (e.g. language differences, challenges associated with brain injury)
- Length of assessments (e.g. participant fatigue)
- Asking sensitive information (e.g. Was this information difficult to divulge?)

What were the work-arounds/solutions that you came up with?

GENERAL QUESTIONS

9) Do you have any suggestions for how things could be improved in this trial?

- What sorts of things do you think ran well during the trial?
- What sorts of things do you think could have improved how the trial was run?
- In the future, what could have been done differently?

10) What do you think is the general level of receptivity in the health system overall to implementing this kind of work e.g. to working with Aboriginal people with brain injuries in the ways undertaken in Healing Right Way (e.g. with a focus on cultural security, Aboriginal Brain Injury Coordinator support)?

- What about in your own health service? (*if applicable*)
- Do you think it would differ between sites and why?

11) Do you have any other comments you would like to share about Healing Right Way and your involvement?

7. Blinded Assessor Interview Schedule

ASSESSOR ROLE QUESTIONS
1) Can you please explain your role as a Blinded Assessor for the Healing Right Way study?

2) Please describe any barriers or facilitators you encountered while undertaking the following aspects of your role:

- Consent process (e.g. checking on next of kin consent)
- Participant follow-up (e.g. easy/difficult to schedule and reasons why)
- Assessment type (e.g. VAS, providing information on hospital admissions, medications)
- Assessment mode (i.e. in-person, Telehealth, phone)
- Documentation / hand over of forms (e.g. How accessible were the assessment forms? How easy did you find it to complete and return the forms? How easy did you find it to use REDCap?)

What were the work-arounds/solutions that you came up with? Can you please give some specific examples? (e.g. extensive attempts to follow up participants)

SITE QUESTIONS
3) What level of support did you receive from the Research Site Contact(s)?
- What kind of support did they give you? Can you please provide specific examples?

4) Please describe what else helped you in your role as a Blinded Assessor.
(e.g. ALO or other Aboriginal community worker [involvement during assessments], community organisations, research staff, other people, training/resources, strategies, circumstances)

When applicable (if assessments completed at participant's home/community)
5) Please describe your experiences of completing assessments in participants' homes.
- How comfortable did you feel people were with you visiting them?
- What steps did you take to ensure a degree of comfort as well as cultural security for participants with the research process involved?
- How comfortable did you feel? Did you ever feel unsafe?
- During assessments, were participants alone or with family?

ROLE PREPAREDNESS QUESTIONS
6) What is your perception of your preparedness for your role in an RCT surrounding Aboriginal people with brain injury? Please comment on things such as training, resources, and support from Healing Right Way staff.

- How much background information relating to the project did you receive, prior to starting your role?
- How much previous experience did you have working with individuals with acquired brain injury?

- Did you have previous experience working with Aboriginal patients/families?
- Did you feel prepared to work with the Aboriginal patients/families? What helped prepare you?
- Please describe any challenges you experienced working with the Aboriginal patient/families? How did your experiences differ from working with non-Aboriginal patients/families?
- How prepared did the hospital appear for the project in terms of accommodating you as an assessor? (e.g. How prepared did you feel the different wards were for your visit? i.e. did you feel welcome; or have to explain why you were there?)
- How did you find the process of completing all of the required training to be able to execute your role? (e.g. FIM training, using REDCap, cultural security of assessment processes, education relating to the project)
- Do you have any examples of how you used your training?
- What support did you receive from Healing Right Way staff?

7) Please describe any other challenges you experienced in this role. Other aspects of your role may have included:

- Time pressures (e.g. How did you manage having this additional role with your current job?)
- Covid-related issues
- Mode of employment (e.g. getting an ABN, ECU contract)
- Communication with participants (e.g. language differences, challenges associated with brain injury)
- Length of assessments (e.g. participant fatigue)
- Asking sensitive information (e.g. Was this information difficult to divulge?)

What were the work-arounds/solutions that you came up with?

GENERAL QUESTIONS

8) Do you have any suggestions for how things could be improved in this trial?

- What sorts of things do you think ran well during the trial?
- What sorts of things do you think could have improved how the trial was run?
- In the future, what could have been done differently?

9) What do you think is the general level of receptivity in the health system overall to implementing this kind of work e.g. to working with Aboriginal people with brain injuries in the ways undertaken in Healing Right Way (e.g. with a focus on cultural security, Aboriginal Brain Injury Coordinator support)?

- What about in your own health service? (*if applicable*)
- Do you think it would differ between sites and why?
- Do you feel the effort and the work-arounds you put into your role as a Blinded Assessor could be implemented into health services? Why or why not?

10) Do you have any other comments you would like to share about Healing Right Way and your involvement?

7. Research Site Coordinator Interview Schedule

RSC ROLE QUESTIONS

1) Please explain your role as a Research Site Contact.
*(Will be referred to as **site coordinator for the study** - this question will be used as an ice breaker to see how the RSC perceived the role)*

2) To what extent was the implementation of the study helped or compromised due to things happening at your site, specifically things like:

- Identifying patients
- Recruitment
- Assessors
- Covid-related issues
- Staff issues i.e. staff turnover
- Documentation
- Collection and storage of data

- What were the work-arounds/solutions that you came up with?

SITE QUESTIONS

3) What level of endorsement or support did you receive from the leadership both associated with the project team and in your own workplace?

- Principal Investigator (PI) at each site
- Hospital staff [e.g. other management at the hospital (regional director, operations manager, stroke liaison coordinator, allied health manager), ALOs]

- What were the barriers/facilitators?

4) How would you describe the culture of your workplace team? i.e. your immediate environment for this research.

5) To what extent do you think hospital staff are aware of any differences in the needs and preferences related to acquired brain injury in Aboriginal people compared to non-Aboriginal people?

- Did you feel prepared to work with the Aboriginal patients/families? What helped prepare you?
- Please describe any challenges you experienced working with the Aboriginal patient/families? How did your experiences differ from working with non-Aboriginal patients/families?

OFF-SITE QUESTIONS

6) What is your perception of the preparedness for the implementation of the study? Please comment on things such as the training, resources, and support from HRW staff.

- Did you get enough training?
- Were there enough resources, including the folder?
- Did you get enough support from HRW staff?
- Do you think the hospital was prepared for the project to get underway?
- Did the HRW staff help to facilitate site readiness?

- What were the barriers/facilitators?

ABIC QUESTIONS

7) What sort of feedback did you receive from participants, their families or staff regarding their experiences with the Aboriginal brain injury coordinator (ABIC)?
  - What were their perceptions of having brain injury coordinator support?
  - Can you describe any specific information you have heard?
  - Can you describe a specific story?

  - What was your perception of having brain injury coordinator support? Please share any story(ies) that relate to your own experience?

8) How well did the intervention (ABIC role, CST) fit with existing work processes and practices? How much did they change work processes that had existed?

Now some questions related to the Aboriginal brain injury coordinator, starting with getting a sense of how much contact you had with them?
  - Please talk about how the ABIC role added to or changed existing processes and practices, and any issues or complications that arose.
  - How was the intervention (ABIC role, CST) integrated (or not) into work processes?
  - How did it interact or conflict with current programs or processes?

GENERAL QUESTIONS

9) What do you think is the general level of receptivity in the health system overall to implementing this kind of work i.e. to working with Aboriginal people with brain injuries in the ways undertaken in Healing Right Way (e.g. with a focus on cultural security, Aboriginal Brain Injury Coordinator support)?
  - What about in your own health service?
  - Do you think it would differ between sites and why?

10) What aspects of the health system overall do you think affected the implementation of the intervention (ABIC role, CST)?

11) Do you have any other comments you would like to share about HRW and your involvement?
