# Supplementary material for: Process evaluation of a randomised controlled trial intervention designed to improve rehabilitation services for Aboriginal Australians after brain injury: the Healing Right Way Trial
Source: BMC Health Serv Res. 2024 Aug 20;24:946. doi: 10.1186/s12913-024-11390-5 (PMC11334317; doi:10.1186/s12913-024-11390-5)

Supplementary Table 1 Summary of process evaluation data sources by components of HRW

|  | **Component of HRW** | | |
| --- | --- | --- | --- |
| **Data source** | **Trial Processes** | **CST** | **ABIC** |
| Project Log | x | x | x |
| Surveys:  *CST attendee surveys (face-to-face and online)*  *Participant ‘Hospital Experience’ survey*  *(Participant ‘ABIC’ survey)* |  | x  x | x  (x) |
| Semi-structured interviews:  *Chief Investigator*  *Project Manager*  *Data Operations Manager*  *ABIC Coordinator*  *ABICs*  *Research Site Contacts*  *Assessors (baseline and blinded)* | x  x  x  x  x  x  x | x  x | x  x  x  x  x  x |
| Observations | x | x | x |
| Review of meeting minutes (project and partner meetings) | x | x | x |


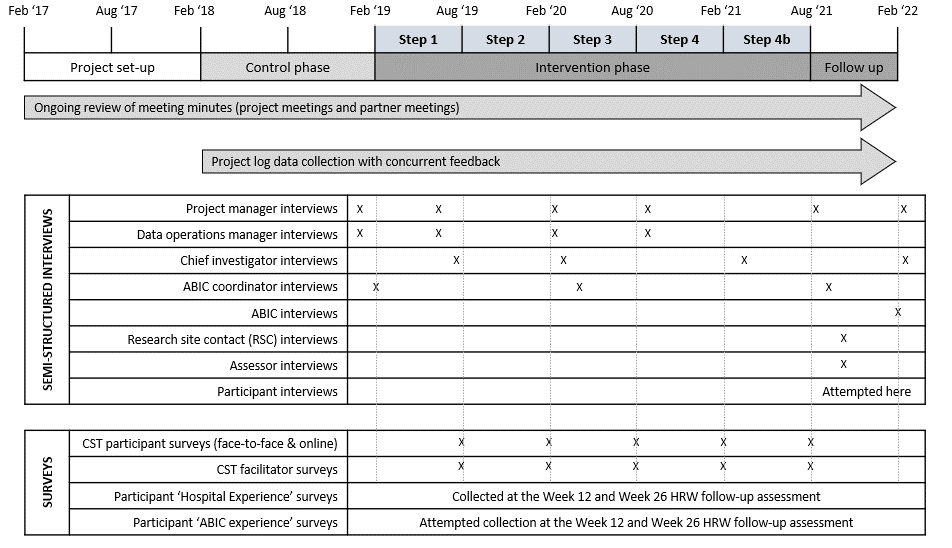
Supplementary Figure 1. Overview of process evaluation data sources and exact time of collection

Supplementary Table 2: Summary of process evaluation findings in relation to HRW trial processes^*^

^*^As the following focused on general research trial processes and research project management rather than the intervention itself, the heading of ‘Intervention’ in comparison CFIR tables has been altered to ‘Research component’ representing the typical research components of an RCT.


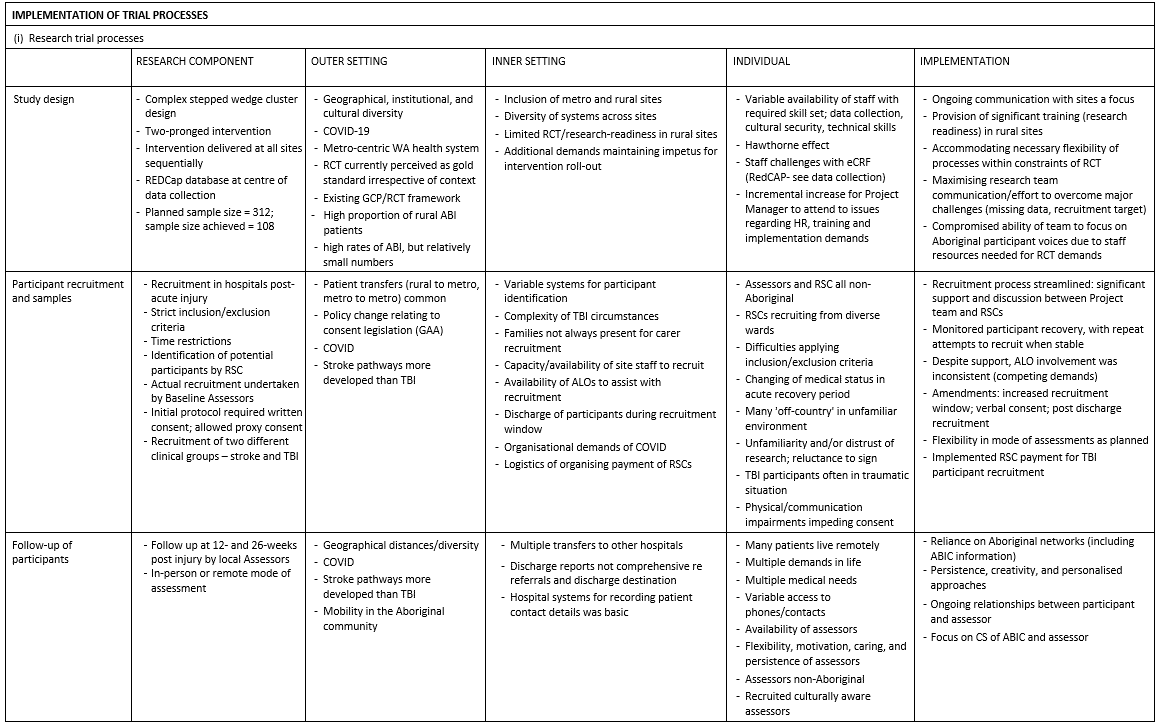


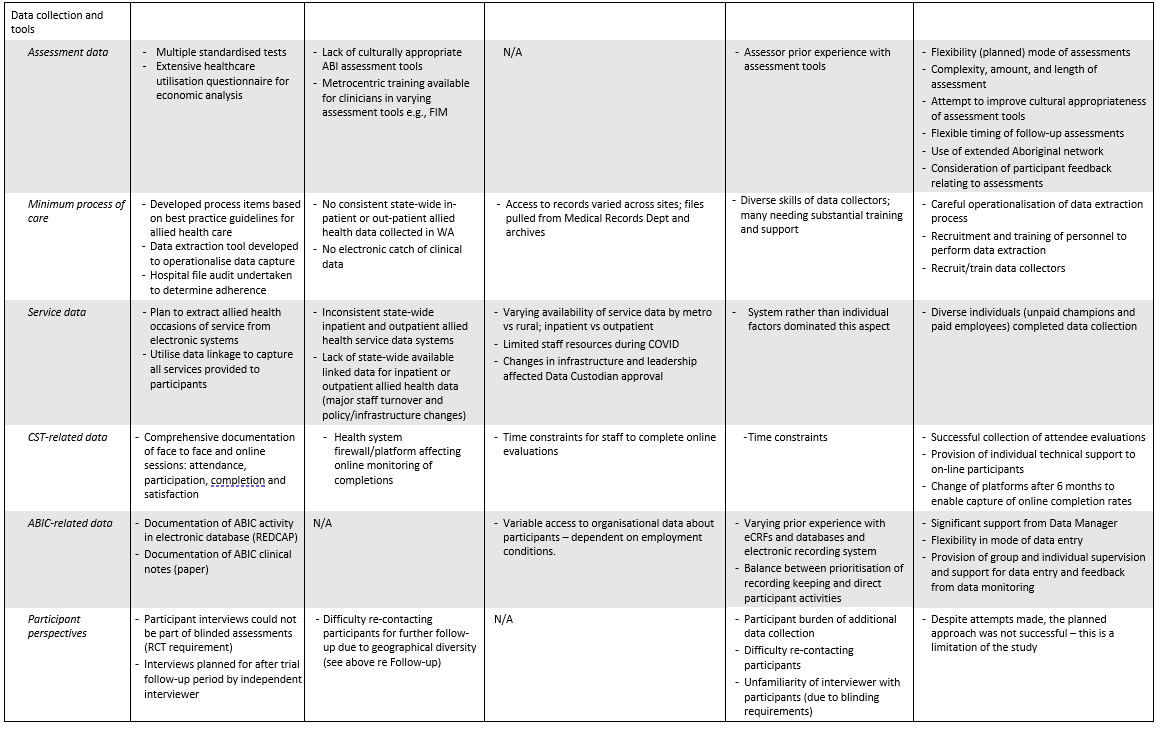


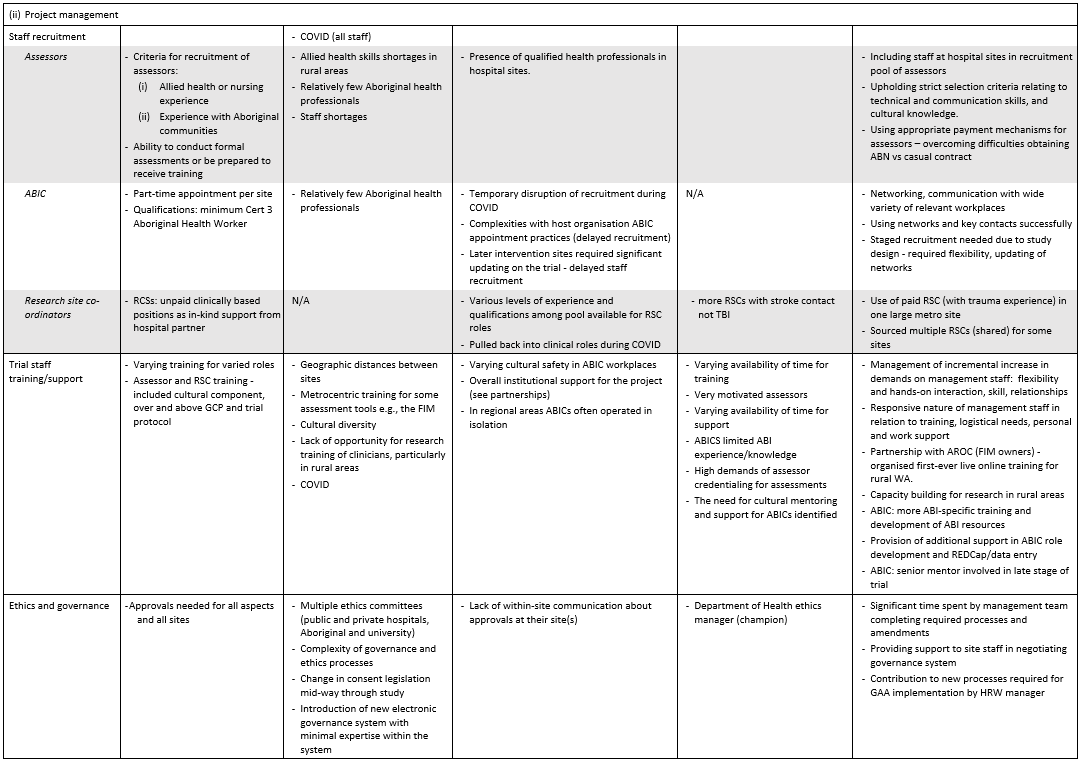


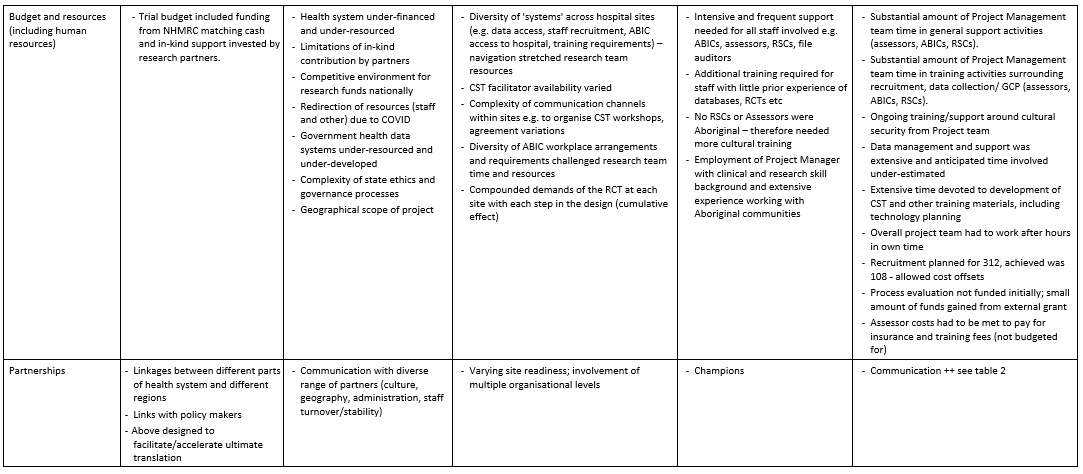


Supplementary Table 3: Summary of process evaluation findings in relation to HRW interventions


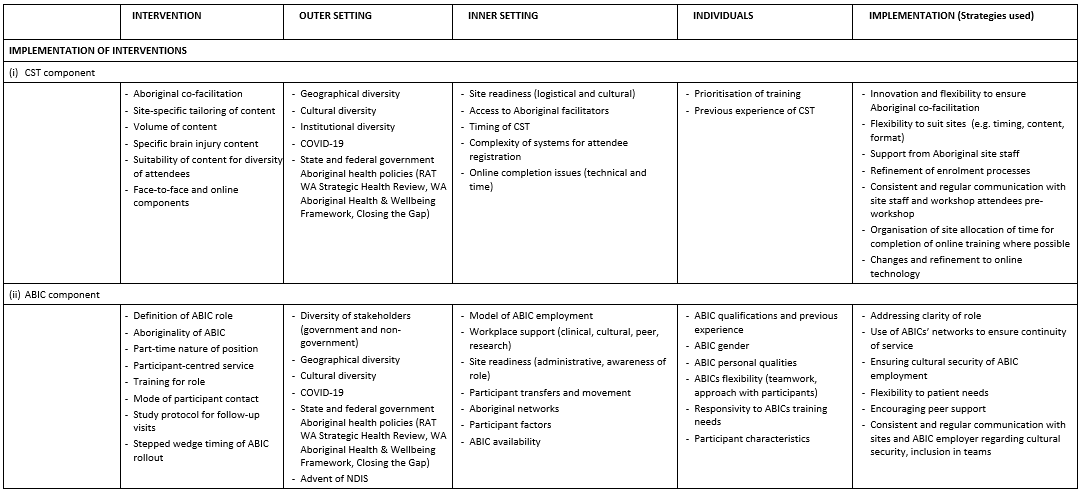

Supplement: Supplementary file 2 — Supplementary Material 2. [file 12913_2024_11390_MOESM2_ESM.docx]
