# Supplementary material for: Process evaluation of a randomised controlled trial intervention designed to improve rehabilitation services for Aboriginal Australians after brain injury: the Healing Right Way Trial
Source: BMC Health Serv Res. 2024 Aug 20;24:946. doi: 10.1186/s12913-024-11390-5 (PMC11334317; doi:10.1186/s12913-024-11390-5)
Supplement: Supplementary file 3 — Supplementary Material 3. [file 12913_2024_11390_MOESM3_ESM.docx]

**Supplementary Table 4: Adaptations to the interventions to address local contexts and challenges, while maintaining fidelity to their original designs**

| **ABIC Adaptations** | **Reason** | **Impact** |
| --- | --- | --- |
| More participant visits done via telephone than face-to-face. | Covid-19. | Participant visits were shorter over the telephone however it allowed for more flexibility. |
| Model of employment (e.g. Hospital, AMS). | Dependent on site preference. | May have affected cultural security of ABIC and also clarity around perception of ABIC role. |
| ‘Sharing’ of case load. | Personal, family, cultural commitments. | Allowed for more flexibility but it meant that ABIC was not always in the same region as the participant. |
| Additional training and support. | ABIC need for more training relating to multiple aspects of their role (brain injury, trial processes and cultural). | Increased ABIC satisfaction and ability to perform job requirements. |
| **CST Adaptations** | **Reason** | **Impact** |
| Change of platform for online content delivery. | Department of Health firewalls prevented access to original platform. | Increased ability to engage with online component of CST. |
| Timing of workshop delivery. | Covid-19. | Fewer workshops than originally intended but still within protocol design (i.e. required number of people attending at each site achieved). |
| More explicit facilitator acknowledgement of the attendee range of experience working with Aboriginal people with brain injury. | On basis of workshop feedback. | Accommodation of participant feedback and potential increased engagement. |
| Increased email follow-up to workshop attendees. | To encourage completion of online content. | Slight improvement in ‘completion’ of training modules. |
